# Comparative analysis of neutrophil dynamics and disease in SARS-CoV-2 Delta and Omicron variants utilizing an *in vivo* feline model for COVID-19

**DOI:** 10.3389/fimmu.2025.1547918

**Published:** 2025-05-22

**Authors:** Sachithra Gunasekara, Shoroq Shatnawi, Sunil More, Breya Ludwig, Sai Narayanan, Miruthula Tamil Selvan, Craig A. Miller, Jennifer M. Rudd

**Affiliations:** ^1^ Department of Veterinary Pathobiology, Oklahoma State University, College of Veterinary Medicine, Stillwater, OK, United States; ^2^ Oklahoma Animal Disease Diagnostic Laboratory, College of Veterinary Medicine, Oklahoma State University, Stillwater, OK, United States

**Keywords:** SARS-CoV-2, COVID-19, Delta, Omicron, immunopathogenesis, neutrophils, NETs, feline

## Abstract

**Introduction:**

The emergence of SARS-CoV-2 variants, particularly Delta (B.1.617.2) and Omicron (XBB.1.5) variants, has substantially influenced the clinical and immunological landscape of COVID-19. This study investigates the differential pathogenicity and immune responses in a feline model infected with these variants, focusing on neutrophil activation, neutrophil extracellular trap (NET) formation, and cytokine profiles.

**Methods:**

Eight pathogen-free cats were inoculated with B.1.617.2 (Delta) SARS-CoV-2 (n=3), XBB.1.5 (Omicron) SARS-CoV-2 (n=3), or vehicle (n=2), and clinical assessments, histopathological examinations, and cytokine analyses were performed post-infection.

**Results:**

Results demonstrate that Delta-infected cats exhibit more severe clinical manifestations characterized by significant elevation in respiratory effort, wheezing, and systemic inflammation compared to Omicron-infected cats, which show milder symptoms, primarily confined to the upper respiratory tract. Histopathological findings suggest pronounced lung damage in Delta-infected cats, whereas Omicron infection resulted in localized pathology. Cytokine profiling demonstrates heightened proinflammatory responses, particularly in Delta-infected cats, characterized by elevated levels of IL-6, IFN-γ and TNF-α while Omicron infection results in less pronounced inflammatory responses. Moreover, neutrophil-related parameters, including total neutrophil counts and banded neutrophils, were significantly elevated in Delta-infected cats, correlating with enhanced NET formation as evidenced by increased NETs-related markers MPO, NE, and citrullinated H3, and NET-specific markers MPO-DNA complexes and cell-free DNA.

**Discussion:**

This study underscores the importance of variant-specific immune responses and highlights the need for targeted therapeutic strategies that mitigate severe lung injury associated with Delta infection, while also considering the distinct immune dynamics observed with the Omicron variant. Furthermore, results support the importance of delineating immune responses concerning future variants. These findings provide valuable insights into the pathogenesis of SARS-CoV-2 in companion animals and inform public health strategies as new variants continue to emerge.

## 1 Introduction

The emergence of severe acute respiratory syndrome coronavirus 2 (SARS-CoV-2) variants substantially impacted the course of the COVID-19 pandemic, influencing disease severity, transmissibility, and vaccine efficacy (1–3). Among these variants, the Delta and Omicron variants have gained significant attention due to their diverse pathogenic profiles and clinical manifestations (1–6). Delta, first identified in late 2020, is associated with more severe clinical outcomes, including higher hospitalization and mortality rates, whereas Omicron, which emerged in late 2021, is characterized by a higher transmission rate but generally less severe disease, particularly in vaccinated individuals (1, 7–9). Omicron primarily affects the upper respiratory tract as opposed to the lower respiratory tract, raising concerns about its transmissibility and potential for immune escape (6, 7, 10). However, the underlying mechanisms driving these variant-specific clinical manifestations remain inadequately understood, especially in animal models that can mimic human COVID-19.

Innate immunity, specifically neutrophils, serves as the first line of defense against pathogens, playing a pivotal role in the immune response to viral infections, including SARS-CoV-2 (11–13). Neutrophils deploy antimicrobial mechanisms, including phagocytosis, reactive oxygen species (ROS) production, and the release of neutrophil extracellular traps (NETs), which are crucial for controlling viral replication and mediating tissue damage during infections (11, 14). Neutrophil extracellular traps (NETs), excessive immune activation, and neutrophil-driven responses have been implicated in severe COVID-19 cases (14–17). NETs are web-like structures composed of DNA strands decorated with antimicrobial proteins, such as myeloperoxidases (MPO) and neutrophil elastases (NE), and are released by activated neutrophils to trap and neutralize pathogens (13, 18–20). However, their overproduction can lead to tissue damage, contributing to lung injury and thromboinflammation (21, 22). Recent studies in human and animal models have indicated that dysregulated NET formation may be a key contributor to COVID-19 severity, predominantly in severe infections with variants such as Delta; emerging evidence suggests that neutrophil responses may vary significantly between different viral variants (10, 23–25). The presence of NET components, such as MPO-DNA complexes and citrullinated histones (Cit-H3), suggest ongoing NETosis, contributing to lung injury through mechanisms that include oxidative stress and tissue remodeling (20, 26). Elevated levels of ROS produced by activated neutrophils can lead to collateral damage to alveolar structures, exacerbating respiratory dysfunction (15, 26). This excessive neutrophilic response, characterized by both systemic inflammation and localized tissue damage, highlights the critical role of neutrophils and NETs in the pathogenesis of severe respiratory disease associated with SARS-CoV-2 infection (16, 21).

Recent studies utilizing animal models, specifically felines, have highlighted the importance of examining immune responses to SARS-CoV-2. Domestic cats exhibit physiological and genetic similarities with humans, making them an excellent model for studying viral pathogenesis and immune dynamics (13, 27–29). Given their susceptibility to SARS-CoV-2, ability to transmit the virus, and potential to develop clinical manifestations mirroring acute COVID-19, understanding cats’ immune responses is critical for both veterinary and public health. Importantly, recent studies regarding the feline COVID-19 model have provided valuable insights into the pathogenesis of infection, depending on different variants of concern (27, 28, 30). Moreover, studies have highlighted the role of neutrophils and NETs in COVID-19 and their contribution to lung injury and systemic inflammation during Delta variant infection in cats (13). The role of neutrophils and NETs in variant-specific immune responses, particularly in feline models, remains underexplored.

This study aims to investigate the differential pathogenicity and immune responses in cats infected with the SARS-CoV-2 Delta and Omicron variants, with a specific emphasis on variant-specific cytokine profiles, histopathological changes, neutrophil activation, and NET formation. By utilizing a feline model, we aim to elucidate variant-specific disease mechanisms and immune dynamics that may provide insights into the distinct clinical outcomes observed in humans infected with these variants. The findings from this study not only enhance our understanding of SARS-CoV-2 pathogenesis in companion animals but also provide broader implications for public health and the management of COVID-19 in humans, by identifying critical immune pathways and potential therapeutic targets as new variants continue to emerge.

## 2 Materials and methods

### 2.1 Virus propagation

Human isolates of the SARS-CoV-2 virus, specifically, B.6.617.2 (Delta variant) and XBB.1.5 (Omicron variant) were acquired from BEI Resources (Manassas, VA, USA) and propagated following established methods (27, 28). Briefly, the viruses were passaged five times on Vero cells (CCL-81; ATCC, Manassas, VA, USA) supplemented with Dulbecco’s Modified Eagle Medium (DMEM, Gibco, Carlsbad, CA, USA), 1% Penicillin-Streptomycin (Gibco, Carlsbad, CA, USA), and 5% fetal bovine serum (Hyclone, Logan, UT, USA). The resulting viral stocks were quantified using the TCID_50_ method, according to the Reed and Muench calculation (31).

### 2.2 Animals, SARS-CoV-2 infection

This research protocol was approved by the Oklahoma State University Institutional Animal Care and Use Committee (IACUC 20–48 STW). A total of eight (female=5, male=3) age-matched (2-years-old) specific pathogen-free (SPF) cats were sourced from an accredited colony at Colorado State University. All cats were acclimatized for thirty days according to standard guidelines, with access to water and food *ad libitum*. Cats were subsequently divided into three groups: cats intended for the infection with Delta variant of SARS-CoV-2 (n=3;2 females, 1 male), cats intended for the infection with Omicron variant of SARS-CoV-2 virus (n=3;2 females, 1 male) and sham-inoculated controls (n=2;1 female, 1 male). The six cats designated for SARS-CoV-2 infection were accommodated at Animal Biosafety Level 3 (ABSL-3) facilities, while the two control cats were housed in standard animal facilities at Oklahoma State University. Comprehensive health assessments including the measurement of body weight, body temperature, and clinical evaluation were conducted prior to infection to confirm their health status.

**Figure 1:**
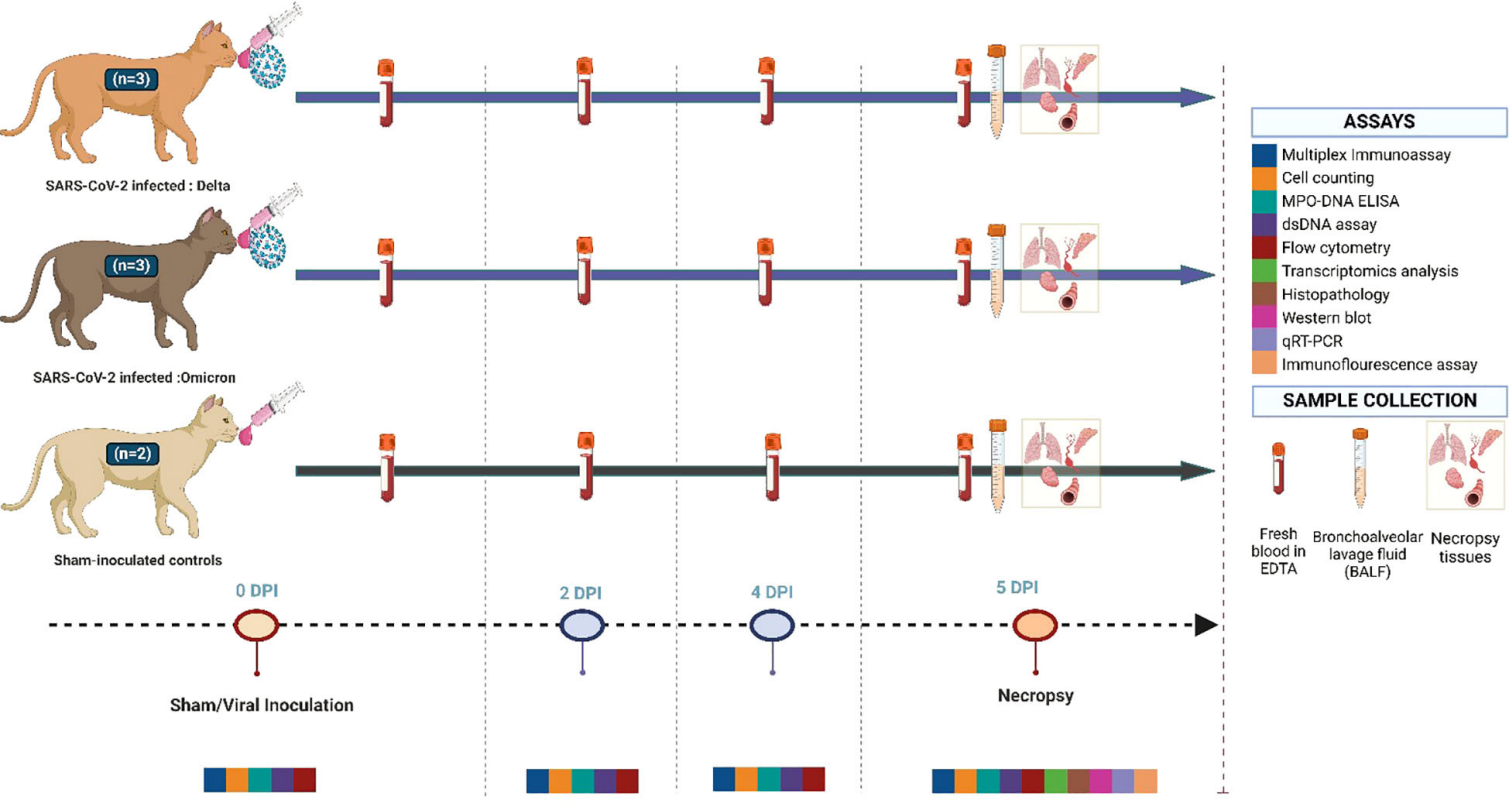
Experimental study design. This illustration outlines the experimental timeline, and the assays conducted throughout the study from a total of eight cats. Three were inoculated with the Delta (B.6.617.2) variant of SARS-CoV-2, three were inoculated with the Omicron (XBB.1.5) variant of SARS-CoV-2, with the remaining two serving as sham-inoculated controls. All cats were euthanized at 5 days post-infection (dpi) following necropsy. Whole blood, bronchoalveolar lavage, and tissues were collected for subsequent analysis. Image made in Biorender.

Following sedation via intramuscular injection of ketamine (4 mg/kg), butorphanol (0.4 mg/kg), and dexmedetomidine (20 µg/kg), six cats were intratracheally and intranasally inoculated with either the Delta variant (n=3) or the Omicron variant (n=3), whereas the remaining two cats were inoculated with DMEM as sham-inoculated controls. Cats (n=6) were inoculated with an equal dose of virus; 0.3 × 10^5^ TCID_50_ SARS-CoV-2 per kg of body weight (Total dose of 1.5 × 10^5^ TCID_50_) using a total volume of 1 ml of DMEM. The controls (n=2) were inoculated with 1 ml of DMEM. All eight cats were humanely euthanized following sedation, using pentobarbital > 80 mg/kg at five days post-infection (dpi) as previously described (13, 27, 28). Following euthanasia, necropsy was conducted for tissue collection (**Figure 1**).

### 2.3 Clinical evaluation

During the course of study, all animals underwent twice daily monitoring using a modified clinical scoring system for felines (**Supplementary Table S1**) as outlined in previous studies (27, 28). Each health parameter, including body temperature, body weight, activity, behavior, respiratory effort, ocular/nasal discharge, coughing, and wheezing, was assessed and scored on a scale from 0 to 3 (0=normal, 1=mild, 2=moderate, 3=severe) by a licensed veterinary practitioner at each selected time point. Individual scores were then compiled to calculate a comprehensive summated clinical score for each animal at each monitored time point.

### 2.4 Sample collection and processing

Following anesthesia at 0-, 2-, 4-, and 5 dpi, 5 ml of whole blood in EDTA was collected from each cat via jugular venipuncture. A portion of the collected blood was reserved for flow cytometry and smear preparation while the remainder was utilized to extract plasma. Plasma was separated by centrifuging at 2000xg for 10 minutes at 4°C and stored at -80°C until further analysis (32). Bronchoalveolar lavage fluid (BALF) was collected from all cats at 5 dpi, pre-euthanasia. In brief, a sterile endotracheal (ET) tube was inserted into the trachea first, and then a catheter tube was introduced via the ET tube. A volume of 20 ml of warm sterile saline solution (Med Vet, IL, USA) was injected into the lungs through the tube. The saline solution was allowed to settle before gently aspirating an equal volume of the fluid back into a conical tube from each cat. Fluid content was centrifuged at 400xg for 7 minutes at 4°C (33, 34). Obtained supernatants were aliquoted into new Eppendorf tubes and stored at -80°C until further analysis. The cell pellets were utilized for flow cytometry. At necropsy, several tissues, including lungs, tonsils, retroperitoneal lymph nodes (RPLN), distal trachea (DT), and nasal turbinates (NT), were collected from all cats. Tissues were subdivided and either preserved at -80°C or collected into tissue cassettes and fixed for 5 days in 10% neutral-buffered formalin before transferring to ethanol for 3 days as previously described (27). A small section of the lung tissue from each animal was placed in a tube with sterile phosphate-buffered saline (PBS, Gibco, Carlsbad, CA, USA) to obtain lung lysates. Briefly, 30–50 mg of lung tissue from each cat was transferred into a 5 ml solution containing DNase I (0.2 µl/ml; Thermofisher, Wilmington, DE, USA) and type IV collagenase (0.5 mg/ml; (Thermofisher, Wilmington, DE, USA) prepared in PBS. The tubes were incubated for 15 minutes at 37°C. Following the addition of an equal volume of PBS, the reaction mixture was consecutively filtered through a 70 µm and 40 µm cell strainer into a 50 ml conical tube while grinding the tissues into a suspension. Following that, 5 mL RBC lysis buffer was added and incubated for 10 minutes at room temperature which was neutralized by the addition of an equal volume of PBS. The solution was centrifuged for 5 minutes at 1500 RPM at 4°C (35). The supernatants from each sample were separated and stored at -80°C till further analysis while the pellets were resuspended in 1 ml of DMEM and utilized for flow cytometry.

Prior to other functional assays, confirmation of SARS-CoV-2 infection and the viral quantification was performed via ddPCR. All six cats were confirmed to be infected, while sham-inoculated controls were negative for SARS-CoV-2 infection.

### 2.5 Histopathology

Selected tissues were processed for histopathology as previously described (27, 28). Briefly, 5 μm thick paraffin sections of tissues were collected onto positively charged slides following hematoxylin and eosin (H&E) staining. H&E staining, microscopic evaluation, and histopathologic scoring of the tissues was performed by a board-certified veterinary pathologist. The tissues were assigned a quantitative histopathological score from 0 to 4 (0=no change, 1=minimal change, 2=mild change, 3=moderate change, 4=marked change) and the necropsy tissues were further evaluated for pathologic lesions as documented in previous human and animal studies on COVID-19 (27, 28, 36–38).

### 2.6 Multiplex immunoassay for cytokine profiling

Systemic and respiratory cytokine concentrations in plasma and BALF samples were measured, using a commercially available feline cytokine-chemokine magnetic bead panel ELISA kit (Millipore-Sigma, Burlington, MA, USA) according to the manufacturer’s instructions and as previously mentioned (13). The assay involved a comprehensive panel of soluble cytokine molecules (s-Fas, IL-1β, SCF, TNF-α, PDGF-BB, IL-18, IFN-γ, GM-CSF,IL-4, IL-2, IL-6, RANTES, FLT-3L,KC,IL-12, MCP-1,IL-13, SDF-1, and IL-8). In brief, 25 μl of the standards, plasma, or BALF was mixed with an equal volume of antibody-coated beads following the addition of assay buffer or serum matrix in a 96-well plate. The plate was then incubated overnight at 4°C on a shaker. The next day, each well was supplemented with 25 μl of biotinylated detection antibodies along with streptavidin-phycoerythrin for signal amplification. Fluorescence measurements and data acquisition were carried out via the Bio-Plex^®^ 200 multiplex detection system (Bio-Rad, CA, USA) and the Bio-Plex manager software (Bio-Rad, CA, USA) respectively.

### 2.7 Lung RNA sequencing and analysis

Lung RNA sequencing was conducted in all cats as previously outlined (13, 27). Briefly, RNA extraction was performed using a QIAGEN RNA isolation kit (Thermofisher, Wilmington, DE, USA). cDNA library preparation and RNA sequencing was executed by Azenta Co Ltd. (South Plainfield, NJ, USA) using llumina^®^ NovaSeq™ with 2x150 base pair configuration, 20–30 million read depth and data quality with ≥80% bases with Q30 or higher. Overall analysis included quality control using fast QC, trimming, mapping, aligning with the reference genome *F.catus_Fca126_mat1.0_*genome sequence file and differentially expressed genes (DEG) analysis. (DEGs) were obtained for controls versus Delta infected, controls versus Omicron infected, and Delta infected versus Omicron infected with Benjamini–Hochberg (BH) adjusted *p*-value < 0.05. Volcano plots were created using the ggplot2 package (v 3.4.4.) in R (v 4.3.2) with the log fold changes on the x-axis and negative logarithm (base 10) of the *p* values on the y-axis for all comparisons. Gene Ontology (GO) and Kyoto Encyclopedia of Genes and Genomes (KEGG) pathway analyses were performed on all comparisons via the online DAVID bioinformatics tools (v 6.8). Of the pathway analysis outcomes, BH-adjusted *p*-value < 0.05 were considered to create the bar plots and bubble plots for enrichment analysis using R packages (v 4.3.2).

### 2.8 Preparation of blood smears and BALF slides, cell counts

Blood smears were prepared for each animal for each time point. Briefly, a drop of blood was placed on one end of a clean glass slide. A second slide was used to produce a thin smear by spreading the slide at a 45° angle to the first slide (39). For the BALF, the cell suspensions were mixed thoroughly by gently inverting the tube and cytocentrifuged as previously described (40). The slides were inserted into the cytocentrifuge funnels (Statspin Cytofuge 2, Beckman Coulter, Inc., CA, USA). A volume of 150 μl was pipetted into the funnel and cytocentrifuged at a speed of 600 rpm for 5 minutes. Processed slides were carefully removed. Both types of slides were air-dried and stained using Kwik-Diff™ staining (Thermofisher, Wilmington, DE, USA) under the manufacturer’s instructions. In brief, the slides were sequentially submerged in fixative solution, eosinophilic stain, and basophilic stain for 15 s each. Following the staining procedures, slides were rinsed gently with distilled water and air dried. Counts for total neutrophils, banded neutrophils, and lymphocytes were acquired via manual cell counting.

### 2.9 Isolation of RNA and proteins from lung tissues

Proteins and total RNA were extracted from the lungs of all cats using TRI-reagent method (MRC, Cincinnati, OH, USA) as per manufacturer’s recommendations and as previously stated (13). Specifically, 50–100 mg of the lung from each cat was homogenized in TRI reagent, followed by phase separation using 1–bromo–3–chloropropane (BCP MRC, Cincinnati, OH, USA). Total RNA was precipitated from the aqueous phase using isopropanol and purified by ethanol washes. The pellets were then air-dried, resuspended in nuclease-free water, and stored at -80°C for subsequent analysis. Proteins were precipitated from the organic phase using acetone washes and purified using consecutive washes with guanidine hydrochloride, ethanol, and glycerol. Air-dried pellets were resuspended in 1% Sodium dodecyl sulfate (SDS) and stored at -20°C until further analysis.

### 2.10 Western blot analysis

Protein contents in the lungs of all cats were quantified using the dye reagent concentrate following manufacturer’s recommendations (Bio-Rad, Hercules, CA, USA) and as previously described (13), using bovine serum albumin as the standard (Bio-Rad, Hercules, CA, USA). Absorbance was measured at 595nm using the SpectraMax M2 microplate reader (Molecular Devices, CA, USA). Based on quantification, proteins (20μg) were prepared on 1X SDS sample buffer (0.06 M Tris (pH 6.8), 2.1% (w/v) SDS, 5% (v/v) glycerol, and 1% (v/v) 2-mercapto-ethanol) and separated on 10% SDS-PAGE alongside a pre-stained protein ladder (ThermoFisher Scientific, Waltham, MA, USA). Following electrophoresis, proteins were transferred onto a nitrocellulose membrane using a semi-dry electro-blotting apparatus (Transblot, Biorad, USA). Prior to immunodetection, membranes were blocked with 5% skim milk for 1 hour at room temperature. Membranes were then incubated with the respective primary antibodies; Myeloperoxidase in 1:500 dilution (MPO, FabGennix, TX, USA), citrullinated histone H3 in 1:1000 dilution (Cit.H3, Abbomax, CA, USA), neutrophil elastase in 1:500 dilution (NE: Invitrogen, Thermofisher, Wilmington, DE, USA), Glyceraldehyde 3-phosphate dehydrogenase in 1:2000 dilution (GAPDH, Abclonals, MA, USA) as the loading control. Membranes were incubated for 1 hour at room temperature with a 1:2000 dilution of horse-radish peroxidase-conjugated goat anti-rabbit secondary antibody (ThermoFisher Scientific, Waltham, MA, USA) following visualization with chemiluminescent peroxidase substrate (ThermoFisher Scientific, Waltham, MA, USA). Images were captured using Amersham Imager 600 (GE Healthcare, Pittsburg, PA) and quantified using Image J software (v 1.8.0). Target protein expression was normalized to GAPDH during analysis.

### 2.11 Quantitative real-time PCR

Quantification and the purification of the total RNA from all cats were determined using NanoDrop ND-1000 (Thermo Fisher Scientific, Wilmington, DE, USA). Equal amounts (1000ng) of RNA from each cat were converted into cDNA using superscript II reverse transcriptase (Invitrogen, Carlsbad, CA, USA) according to manufacturer’s guidelines. qRT-PCR was performed using the SYBR green system (Thermo Fisher Scientific, Wilmington, DE, USA) as previously described. In brief, the reaction mixture (20 μl) consisted of 10 μl 2X SYBR green PCR master mix, 5μl of the cDNA template,1 μl of 10 μm previously published (13) forward and reverse primers of the gene of interest (MPO, NE, and Histone 3) and 4 μl nuclease-free water. The PCR reaction was performed using QuantStudio 6 Pro Real-Time PCR Systems (Applied Biosystems, Carlsbad, CA, USA) on a 96-well plate, according to manufacturer’s recommendations and the validated thermal cycle (initial denaturation step at 95°C for 10 min, followed by 40 cycles at 95°C for 15s, 60°C for 60s, followed by thermal dissociation at 95°C for 15s, 60°C for 60s, and 95°C for 1s). Relative expression and the fold change was determined by following the previously outlined methods (41), using GAPDH as the standard control.

### 2.12 Immunofluorescence assay

Immunofluorescence Assay was performed on both lung tissues and BALF slides of all cats using previously outlined methods (13, 27). For the formalin fixed paraffin-embedded lungs, the slides were initially rehydrated through a series of toluene and ethanol changes followed by antigen retrieval using the Citrate unmasking solution (Cell Signal, Danvers, MA, USA). For the cytocentrifuged BALF slides, samples were fixed with 4% paraformaldehyde (4% PFA, Thermofisher Scientific, Wilmington, DE, USA) for 10 minutes at room temperature. Afterwards, the permeabilization buffer was pipetted onto the slides and incubated for 5 minutes at room temperature. The subsequent steps were consistent for both BALF and lung tissues, with variations in buffer usage as previously described (27, 42). Following one hour blocking step at room temperature, slides were incubated with respective primary antibodies; MPO (1:200, Thermofisher, Wilmington, DE, USA), NE (1:200, Invitrogen, Thermofisher, Wilmington, DE, USA) and Cit. H3 (1:200, Abbomax, CA, USA) overnight at 4°C. Anti-rabbit Alexa fluor-555 secondary antibody (Cell Signaling, Danvers, MA, USA) was added on the following day, while using 4’,6-diamidino-2-phenylindole (DAPI, Cell Signaling, Danvers, MA, USA) as the nuclear counterstain. Slides were coverslipped using Epredia™ Immu-Mount™ (Thermofisher Scientific, Wilmington, DE, USA) and the images were acquired with Zeiss LSM 980 Airyscan 2 confocal laser scanning microscope, later analyzed using ZEN blue software (v.1.10).

### 2.13 MPO-DNA ELISA

MPO-DNA enzyme-linked immunosorbent assay (ELISA) was utilized to quantify the MPO-DNA complexes in plasma, BALF, and lung lysates as previously described (13). Briefly, 100 μl of rabbit anti-myeloperoxidase poly-clonal antibody (1:1000 dilution, Thermofisher, Wilmington, DE, USA) was coated onto a 96-well plate and incubated overnight on a plate shaker at 4°C. The plate was then blocked with 1% bovine serum albumin (Millipore-Sigma, Burlington, MA, USA) in PBS for 2 hours at room temperature. A volume of 100 μl of each sample, premixed with a peroxidase-labeled anti-DNA detection antibody (Cell Death Detection ELISA kit, Roche, Milli-pore-Sigma, Burlington, MA, USA) and diluted at 1:40 in the incubation buffer was then added to the wells. The immunoreaction was developed by adding 100 μl of 2,2’-azino-bis-(3-ethylbenzothiazoline-6-sulphonic acid) (ABTS) substrate and terminated with ABTS stop solution. The optical density of each well was measured at 405 nm using SpectraMax M2 microplate reader.

### 2.14 Quant-iT PicoGreen dsDNA assay

Cell-free DNA in plasma, BALF, and lung lysates were quantified utilizing commercially available Quant-iT Pico Green dsDNA assay kit (Thermofisher, Wilmington, DE, USA), under manufacturer’s instructions. In brief, 100 μl of each sample diluted in TE buffer was added onto a 96-well plate. Then, 100 μl of the Quant-iT™ PicoGreen™ dsDNA reagent was added onto the samples and incubated for 5 minutes at room temperature. Fluorescence was measured using a microplate reader (Cytation-5, Agilent, Santa Clara, CA, USA) at recommended wavelengths (excitation ∼480 nm, emission ∼520 nm).

### 2.15 Flow cytometry

Flow cytometry was performed on all animals using blood samples collected at 0-, 2-, 4-, and 5 dpi, and on BALF and lung lysates collected at 5 dpi. Cell counts were obtained from cell pellets of both BALF and lung samples of all cats following the addition of 1–2 million cells to FACS tubes. For whole blood, 50 μl of blood was added into each tube. The staining procedure for BAL cells, whole blood, and lung cells involved the addition of the antibodies of interest, namely CD11b (PE, RnD biosystems, NE, MN, USA), CD44 (BV450, BD-Sciences, NJ, USA), CD14 (BV-515, Bio-Rad, Hercules, CA, USA) CXCR2 (PerCP, Thermofisher, Wilmington, DE, USA), CXCR4 (AF750, RnD biosystems, NE, MN, USA) and CCR5 (APC, RnD biosystems, NE, MN, USA)under manufacturer’s recommendations which was then incubated for 20 minutes in the dark at 4°C. Blood samples included an additional step in which the red blood cells were lysed using the IMMUNOPREP Reagent System (Beckman coulter, CA, USA). Data from all samples were acquired using BD FACSAria II software (Diva9.0.1., San Jose, CA, USA) and analyzed with FlowJo software (v.10.8.0.) (Ashland, OR, USA) using the gating strategy shown in **Supplementary Figure S2**. Uniform Manifold Approximation and Projection (UMAP) algorithm was implemented in OMIQ online software (Dotmatics, Boston, MA, USA) for BAL cells, lung cells, and blood at 5 dpi to visualize unbiased immune cell clusters.

### 2.16 Statistical analyses

Statistical analyses were conducted using GraphPad Prism (V10.1.3). Nonparametric data were expressed as mean ± SEM. Either one-way or two-way ANOVA, followed by Fisher’s Least Significant Difference (LSD) test were employed for multiple group comparisons in which a *p*-value of 0.05 or less was considered statistically significant.

## 3 Results

### 3.1 Clinical evaluation revealed increased disease progression in Delta-infected cats compared to Omicron-infected cats

**Figure 2:**
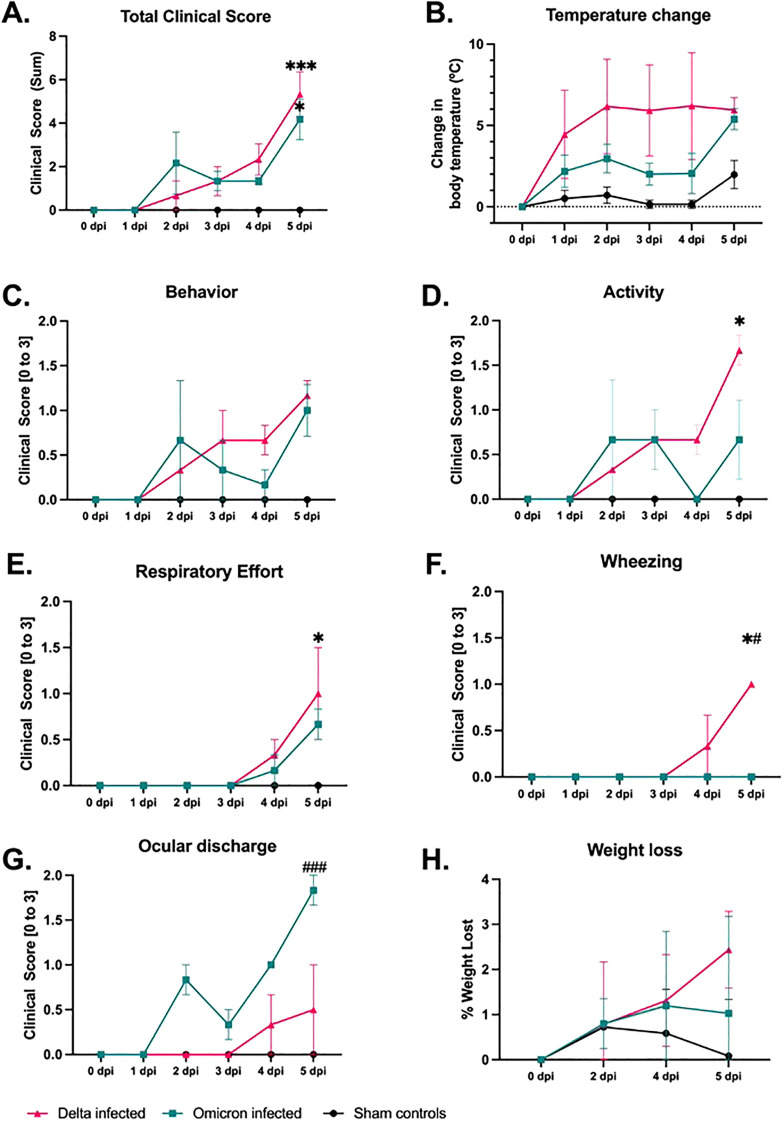
Comparative analyses of clinical parameters in cats infected with Delta or Omicron variants of SARS-CoV-2 and sham-inoculated controls. The figure illustrates the temporal progression of various clinical parameters recorded from a total of 8 cats: sham-inoculated controls (n=2), Delta-infected (n=3), and Omicron-infected (n=3). **(A)** Summated clinical scores for each group indicate significantly worsened clinical severity in all infected cats versus sham-inoculated controls. **(B-H)** provides the variation in individual clinical parameters. across the three groups over time: **(B)** Core temperature changes, **(C)** behavioral changes, **(D)** activity levels, **(E)** respiratory effort, **(F)** wheezing, **(G)** ocular discharge, and **(H)** percentage weight loss. Both variants of concern demonstrated significant disease progression compared to sham-inoculated controls, with Delta-infected cats causing the most severe overall progression. Statistical comparisons were conducted via two-way ANOVA and the data are represented as mean ± SEM. Statistical significances compared to sham controls are indicated by *p < 0.05, and ***p < 0.001 while comparisons between Delta versus Omicron variants are represented by ^#^p < 0.05, and ^###^ p < 0.001.

The clinical assessment of SARS-CoV-2 infected cats revealed significant differences between Delta and Omicron variants. Summated clinical scores (**Figure 2A**) indicate significant disease progression in both variants at 5 dpi compared to controls (p < 0.05, p < 0.001). Moreover, Delta-infected cats demonstrate a non-significant increase in clinical parameters including changes in body temperature (**Figure 2B**), behavior (**Figure 2C**) and significant changes activity (**Figure 2D**), respiratory effort (**Figure 2E**), and wheezing (**Figure 2F**) compared to controls (p < 0.05). In contrast, ocular discharge (**Figure 2G**) was significantly higher in Omicron-infected cats by 5 dpi (p < 0.001) which suggests variant-specific manifestations, specifically in the upper respiratory system. Additionally, Delta-infected cats showed a non-significant trend towards a greater weight loss (**Figure 2H**). Overall, the findings suggest more severe clinical manifestations in Delta-infected cats compared to Omicron-infected cats and sham-inoculated controls.

### 3.2 Histopathological and gross examination revealed differential multi-organ pathology in Delta versus Omicron infected cats

**Figure 3:**
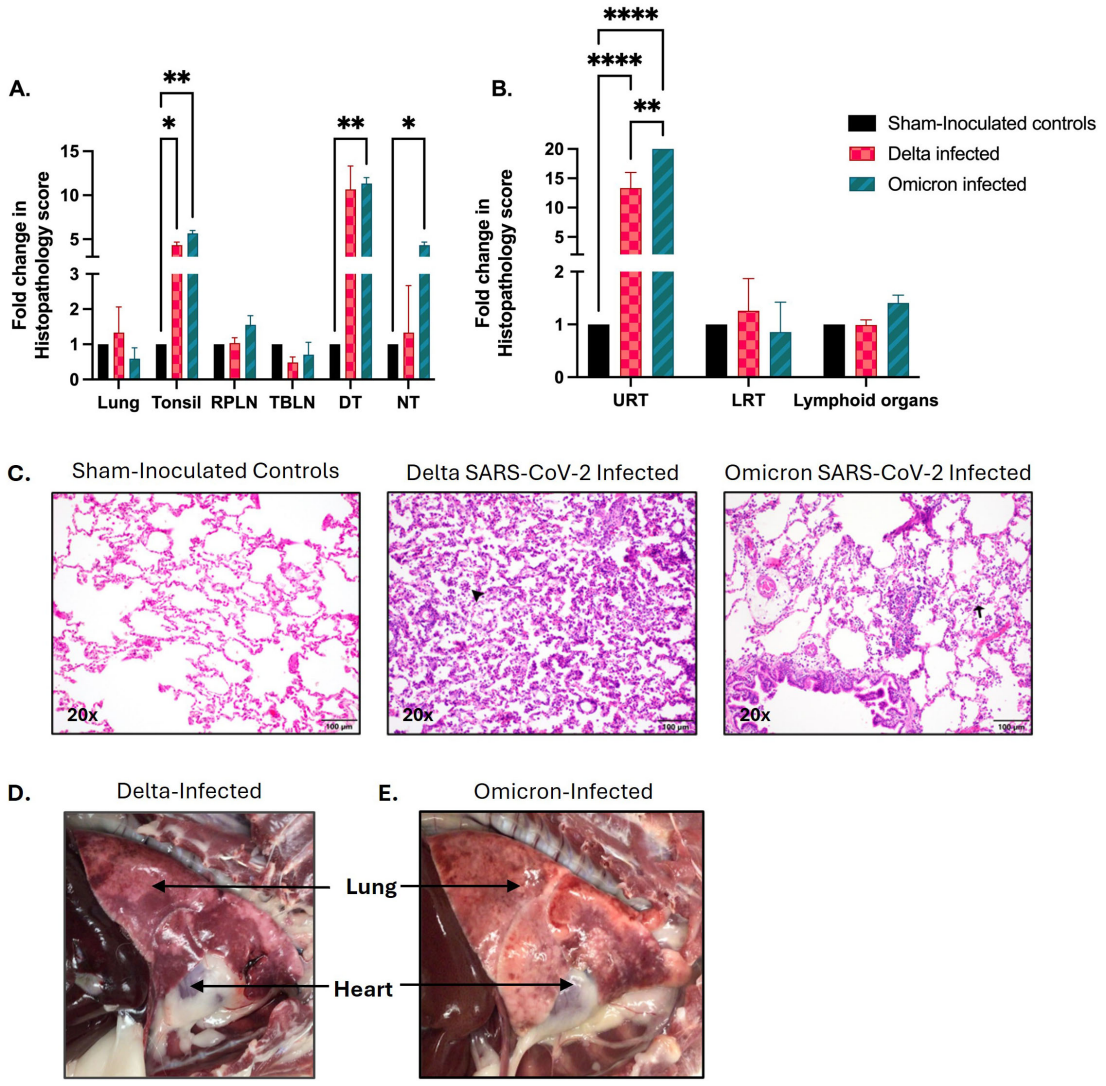
Pathological analysis of SARS-CoV-2 infected Delta and Omicron cats compared to sham controls. The figure illustrates both quantitative and qualitative analyses of tissue pathology following infection with different SARS-CoV-2 variants of concern. **(A)** represents quantitative histopathological scoring for each individual tissue (Lung, tonsil, retropharyngeal lymph node (RPLN), tracheobronchial lymph node (TBLN), distal trachea (DT), and nasal turbinate (NT). Both Delta and Omicron-infected groups exhibit significant changes in pathology scores across multiple tissues. **(B)** represents the quantitative histopathological scoring for grouped regions namely upper respiratory tract (URT), lower respiratory tract (LRT), and lymphoid organs. Both variants showed significantly higher scoring in the URT compared to the control group, with Omicron-infected cats exhibiting a notable increase compared to Delta-infected cats. **(C)** displays H&E stained lung sections from cats infected with Delta and Omicron variants of SARS-CoV-2, as well as sham-inoculated controls. Sham-inoculated controls exhibit intact alveolar architecture, with thin septa and clear alveolar spaces. Delta-infected lungs display the most severe architectural damage, including substantial septal thickening, heavy inflammatory infiltration of neutrophils and macrophages (black arrowhead), alveolar collapse, and consolidation, indicating extensive tissue damage and impaired lung function. In contrast, Omicron-infected lungs show mild septal thickening and moderate neutrophil infiltration (black arrow), with partial disruption of the alveolar structure. **(D, E)** demonstrates the gross pathology of the lungs from SARS-CoV-2 **(D)** Delta and **(E)** Omicron-infected cats. Images highlight the greater severity of lung pathology in Delta-infected cats compared to those infected with the Omicron variant. Statistical significance was assessed using two-way ANOVA, and p-values are indicated as follows: *p < 0.05, **p < 0.01, ****p < 0.0001 with data represented as mean ± SEM. Magnification: **(C)** 20x, scale bar = 100 µm.

The histopathological analysis shown in **Figure 3** provides a comparative assessment of tissue damage in cats infected with the SARS-CoV-2 Delta and Omicron variants, relative to sham-inoculated controls. In **Figure 3A**, the fold change in histopathology scores shows significant differences across multiple tissues between Delta- and Omicron-infected cats compared to sham-inoculated controls. Delta-infected cats demonstrated a nonsignificant increase in lung histopathology scores. Both Delta (p < 0.05) and Omicron (p < 0.01) infections led to significantly higher pathology scores in the tonsils compared to controls. Omicron infection also resulted in significantly elevated pathology in distal trachea (p < 0.05), and nasal turbinates (p < 0.01) compared to controls. Both variants induced mild changes in the retropharyngeal lymph node, although the differences were not statistically significant. As shown in **Figure 3B**, both Delta and Omicron-infected cats exhibited significant pathology in the upper respiratory tract (URT), with Omicron-infected cats showing a notably higher fold change compared to Delta-infected cats and sham controls (p < 0.0001). Pathology in the lower respiratory tract (LRT) and lymphoid organs was elevated for both variants compared to controls, with Delta-infected cats showing a nonsignificant increase in lower respiratory tissue damage compared to Omicron-infected cats and sham controls.

The analysis of H&E-stained lung tissue sections (**Figure 3C**) from cats infected with SARS-CoV-2 Delta and Omicron variants and sham-inoculated controls, revealed distinct patterns of lung injury (**Supplementary Figure S3**). The sham-inoculated control lungs showed normal histology with intact alveolar septa, clear alveolar spaces, and no signs of inflammation, serving as a baseline for comparison. In contrast, the omicron-infected lungs display mild to moderate alveolar septal thickening and some inflammatory cell infiltration, with partial disruption of the normal lung architecture indicating localized inflammation and mild lung injury. Importantly, Delta-infected lungs exhibited severe pathology, including significant septal thickening, extensive inflammatory infiltration, and alveolar collapse. The widespread consolidation observed suggests impaired lung function and severe respiratory involvement. In summary, while both variants cause lung damage, Delta infection resulted in more extensive and severe tissue damage compared to Omicron, with greater disruption of lung architecture and more pronounced inflammation. The gross pathology of the lungs from Delta and Omicron-infected cats revealed distinct differences in the severity of tissue damage. Lungs of Delta-infected cats (**Figure 3D**) exhibited severe consolidation, with dark red to purple discoloration indicating widespread congestion, hemorrhage, and significant tissue damage. The firm and spotted patches indicate areas of necrosis and inflammation, likely leading to impaired lung function and severe respiratory distress. In contrast, the Omicron-infected cats (**Figure 3E**) showed moderate consolidation, with patchy areas of red discoloration indicating localized congestion and hemorrhage which is far less extensive compared to the delta-infected lung, with more of the lung tissue retaining its normal appearance.

### 3.3 Differential cytokine profiles are evident in cats infected with Delta and Omicron variants

**Figure 4:**
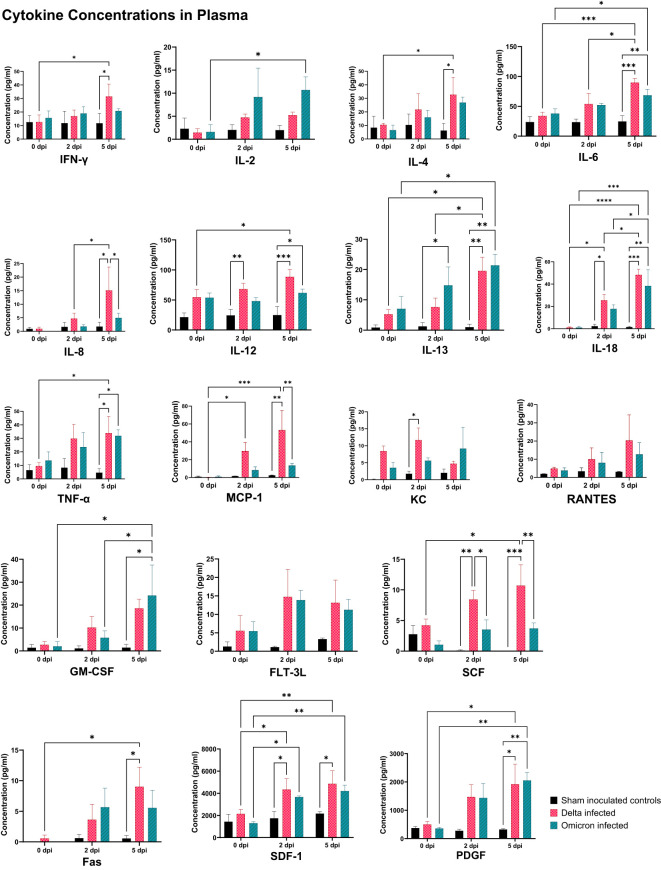
Cytokine and chemokine profiles in plasma of cats infected with SARS-CoV-2 Delta and Omicron variants. This figure demonstrates cytokine concentrations in plasma collected at 0 dpi, 2 dpi, and 5 dpi from all eight cats. Significant alterations were observed in both variants throughout the time points compared to sham inoculated controls. Delta-infected cats showed higher expression levels of several key proinflammatory mediators including IFN-γ, IL-6, IL-8, and TNF-α, particularly at 5 dpi. Statistical analyses were performed using one-way ANOVA and data are presented as mean ± SEM (*p < 0.05, **p < 0.01, ***p < 0.001, ****p < 0.0001).

Our analysis revealed diverse patterns of cytokine expression in the plasma of cats infected with SARS-CoV-2 Delta and Omicron variants at 0-, 2-, and 5-days post-inoculation (dpi), along with sham-inoculated controls (**Figure 4**). Several cytokines including IFN-γ, IL-6, IL-8, TNF-α, MCP-1, IL-4, IL-12, IL-13, SCF, SDF-1, and PGDF were significantly elevated in Delta-infected cats compared to sham controls at 5 dpi (p < 0.05, p < 0.01, p < 0.001). In contrast, Omicron-infected cats showed less pronounced changes in cytokine levels at 5 dpi with an increase in certain cytokines including IL-6, TNF-α, GM-SCF, IL-12, IL-13, and PGDF compared to sham controls (p < 0.05, p < 0.01, p < 0.001). Comparisons between Delta and Omicron variants at 5 dpi revealed significantly higher expression of cytokines including IL-6, IL-8, MCP-1, and SCF in Delta-infected cats compared to Omicron-infected cats (p < 0.05, p < 0.01). In addition, significant elevations were observed at 2 dpi in IL-18, IL-12, and SDF of the Delta-infected cats, while IL-13 was significantly increased in Omicron cats compared to controls (p < 0.05, p < 0.01). SCF was significantly increased compared to both Omicron-infected cats and sham controls (p < 0.05, p < 0.01). Within the Delta group, comparisons between 0 dpi and 2 dpi revealed significant increase in IL-18, SDF-1, and MCP-1 (p < 0.05). Further, the comparison of 0 dpi versus 5 dpi showed significant elevations in IFN-γ, IL-6, IL-18, TNF-α, MCP-1, IL-4, IL-12, IL-13, SCF, SDF-1, Fas, and PDGF (p < 0.05, p < 0.01, p < 0.001). Comparisons between 2 dpi and 5 dpi showed significant increase in IL-6, IL-8, IL-18, and IL-13 (p < 0.05). Within the Omicron group, significant increase in IL-13, IL-2, and GM-CSF was observed at 5 dpi when compared to 0 dpi (p < 0.05).

**Figure 5:**
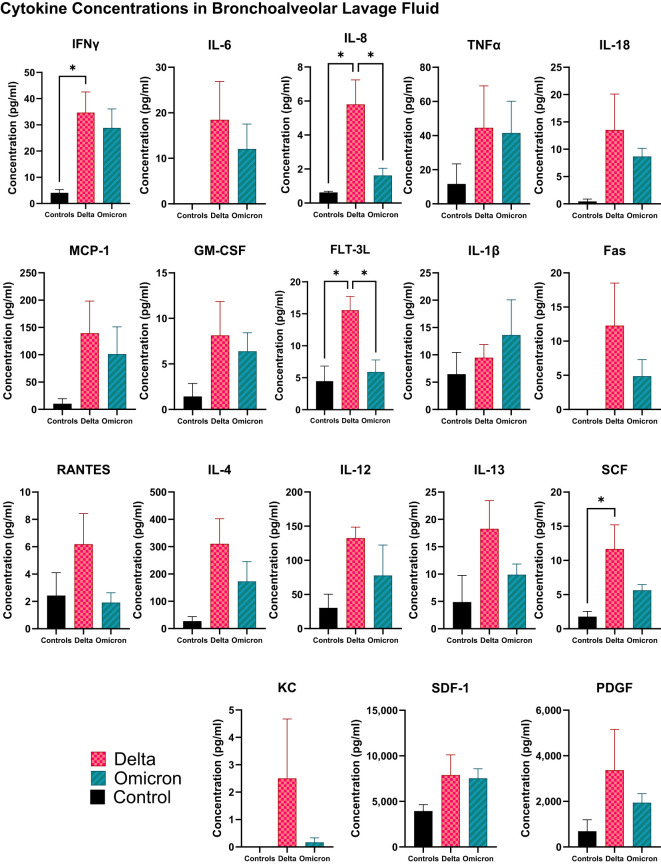
Cytokine and chemokine profiles in BALF of cats infected with SARS-CoV-2 Delta and Omicron variants. This figure illustrates the cytokine concentrations in BALF collected at 5 dpi from all cats. Significant alterations were observed in both variants compared to sham inoculated controls with the Delta-infected cats demonstrating a more pronounced increase in all the cytokines.Statistical analyses were performed using one-way ANOVA and data are presented as mean ± SEM (*p < 0.05).

Overall, the cytokine profiling of BALF (**Figure 5**) exhibited consistently elevated cytokine levels in the Delta-infected cats compared to both Omicron-infected cats and sham controls. Specifically, IFN-γ, IL-8, FLT-3L, and SCF showed significant increase in the Delta-infected cats compared to controls (p < 0.05) with non-significant elevation in other cytokines including IL-6, TNF-α, IL-18, MCP-1, GM-SCF, Fas, RANTES, IL-4, IL-12, IL-13, KC, SDF-1, and PDGF compared to both Omicron infected cats and controls. Furthermore, IL-8, and FLT-3L were significantly elevated in Delta-infected cats compared to Omicron-infected cats (p < 0.05). In contrast, IL-1β was not significantly increased in Omicron-infected cats compared to both Delta-infected cats and control cats.

### 3.4 Distinct molecular and immune pathways are activated in cats infected with Delta and Omicron variants of SARS-CoV-2

**Figure 6:**
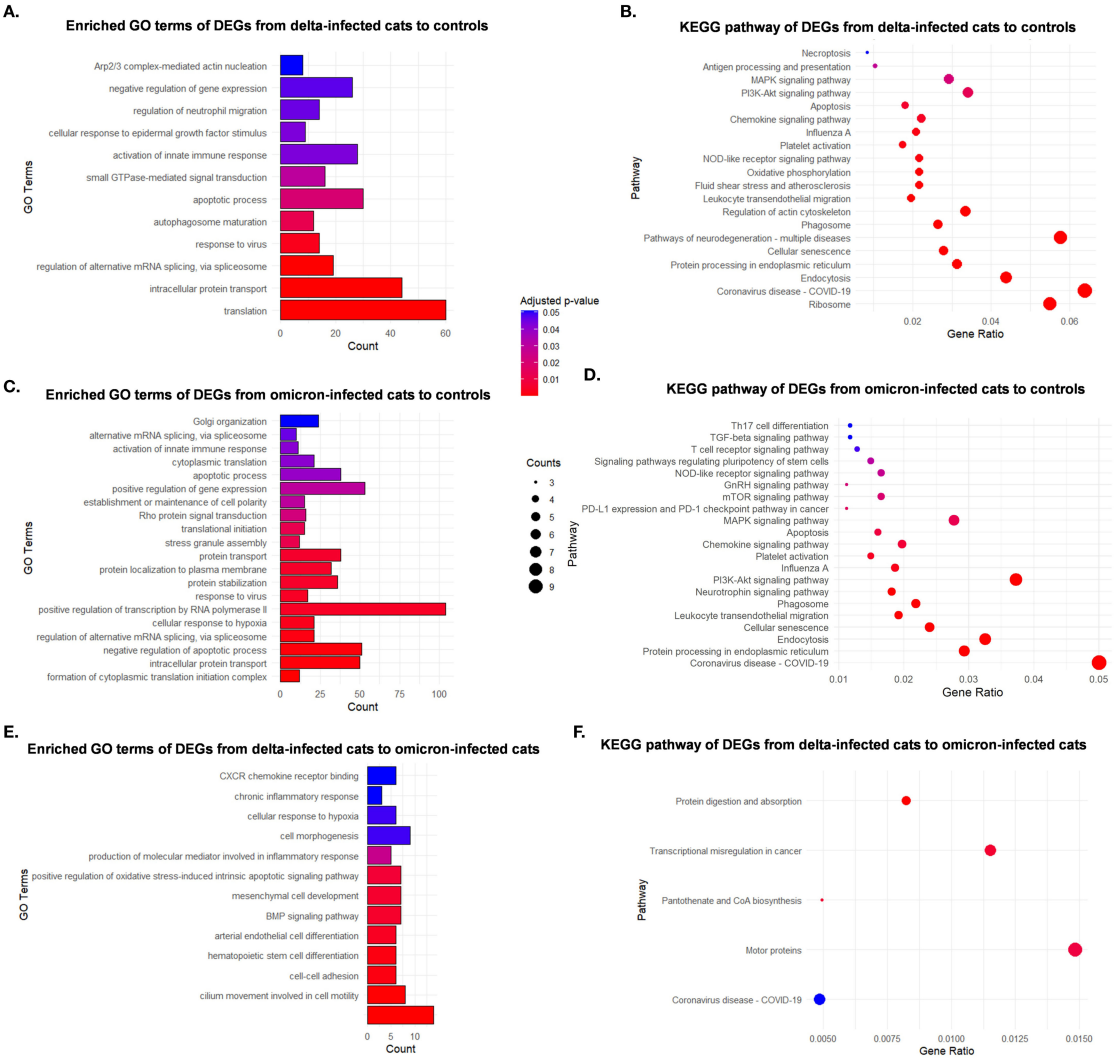
Enrichment in Gene Ontology (GO) terms and Kyoto Encyclopedia of Genes and Genomes (KEGG) pathway analyses of differentially expressed genes (DEGs) in SARS-CoV-2 infected cats compared to controls, and between Delta and Omicron variants. GO and KEGG pathway enrichment analyses show the differential molecular response of cats infected with Delta and Omicron variants of SARS-CoV-2. **(A)** Bar plots of enriched GO terms, **(B)** Bubble plot of top enriched KEGG pathways for DEGs from Delta-infected cats compared to sham-inoculated controls. **(C)** Bar plots of enriched GO terms, **(D)** Bubble plot of top enriched KEGG pathways for DEGs from Omicron-infected cats compared to sham-inoculated controls. **(E)** Bar plots of enriched GO terms, **(F)** Bubble plot of top enriched KEGG pathways for DEGs from Delta-infected cats compared to Omicron-infected cats.

Analysis of RNA sequencing from the lungs of all cats revealed the enriched GO terms and KEGG pathways of DEGs from SARS-CoV-2 infected cats, specifically comparing Delta-infected, Omicron-infected, and control groups. DEG analysis in Delta-infected cats compared to controls resulted in significant upregulation of 1466 genes. DEG analysis in Omicron-infected cats compared to controls revealed significant downregulation of 230 genes and upregulation of 1881 genes. DEG analysis in Delta-infected cats compared to Omicron-infected cats revealed significant downregulation of 325 genes and upregulation of 280 genes. Notable GO terms that were enriched in Delta-infected cats compared to controls (**Figure 6A**) included intracellular protein transport, translation, apoptotic process, regulation of neutrophil migration, activation of innate immune responses, and immune responses to viral stimuli, highlighting key biological processes activated in response to infection. Similarly, KEGG pathways (**Figure 6B**) showed enrichment of the DEGs related to COVID-19 disease and other inflammatory mechanisms including necroptosis, antigen processing and presentation, and MAPK signaling. Omicron-infected cats showed enrichment in GO terms (**Figure 6C**) including activation of innate immune responses, protein localization to the endoplasmic reticulum, response to stress, and regulation of mRNA splicing while KEGG Pathways (**Figure 6D**) revealed involvement of T-cell receptor signaling, several cellular signaling pathways including MAPK, PI3K-Akt, mTOR, and COVID-19 pathways compared to controls. Delta versus Omicron-infected cats revealed differentially regulated GO terms (**Figure 6E**) including chronic inflammatory response, cellular motility, oxidative stress, cell adhesion, and KEGG pathways (**Figure 6F**) enriched in processes including coronavirus disease-COVID-19, protein digestion and absorption, and motor proteins.

### 3.5 Significant difference occurs in neutrophil counts, neutrophil-to-lymphocyte ratio, and banded neutrophils in cats infected with Delta and Omicron variants of SARS-CoV-2

**Figure 7:**
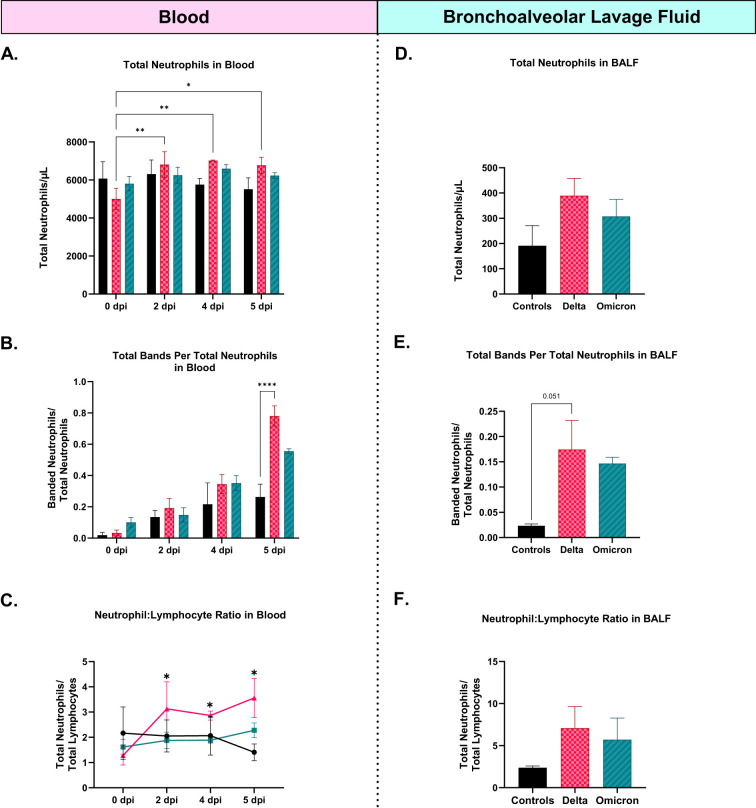
Comparative assessment of neutrophil-related parameters, including total neutrophil counts, neutrophil-to-lymphocyte ratio (NLR), and banded neutrophil counts in cats infected with Delta and Omicron variants of SARS-CoV-2, with sham controls. **(A-C)** demonstrates neutrophil counts, banded neutrophils, and NLR obtained from blood smears prepared from all eight cats throughout the study at 0 dpi, 2 dpi, 4 dpi, and 5 dpi while **(D-F)** provides neutrophil counts, banded neutrophils, and NLR obtained from BALF of all cats at 5 dpi, respectively. Delta-infected cats indicated an increase in all three parameters in both blood and BALF with significant alterations in total neutrophil counts of blood. Statistical comparisons were performed using two-way and one-way ANOVA and data is represented as mean ± SEM (*p < 0.05, **p < 0.01, ****p < 0.0001).

In this analysis, neutrophil-related parameters, including total neutrophil counts, banded neutrophils, and NLR, were evaluated in cats infected with SARS-CoV-2 Delta and Omicron variants, along with sham controls. Blood smears collected at 0, 2, 4, and 5 dpi revealed distinct neutrophil dynamics between the groups. Total neutrophil counts in the Delta-infected cats were significantly elevated at 2 dpi, 4 dpi, and 5 dpi compared to baseline (0 dpi), (p < 0.05, p < 0.01-) (**Figure 7A**). Banded neutrophils in blood were significantly increased in Delta-infected cats at 5 dpi compared to controls (p < 0.01) (**Figure 7B**). NLR in blood (**Figure 7C**) was significantly increased in Delta-infected cats at 2 dpi, 4 dpi, and 5 dpi when compared to their baseline ratios at 0 dpi (p < 0.05). Total neutrophils (**Figure 7D**), banded neutrophils (**Figure 7E**), and NLR (**Figure 7F**) of BALF were increased in Delta-infected cats compared to both Omicron-infected cats and controls non-significantly.

### 3.6 Increased neutrophil activation and expression of related markers are present in Delta-variant-infected cats

**Figure 8:**
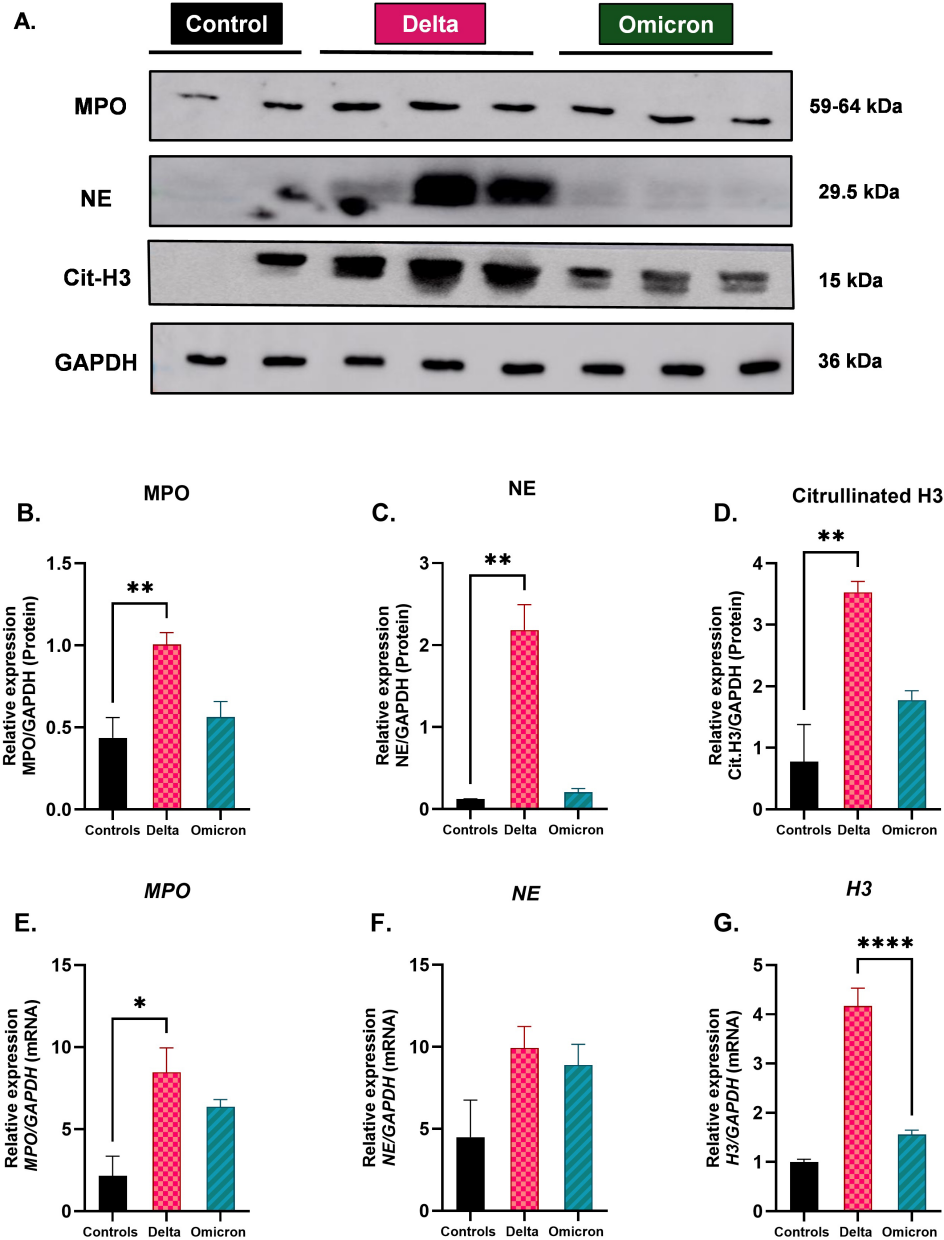
Evaluation of expression in neutrophil activation and NETs-related markers in cats infected with Delta and Omicron variants of SARS-CoV-2. **(A)** Representative western blot image of MPO, NE, and citrullinated histone H3 in lung tissues of all eight cats. GAPDH serves as a loading control. **(B-D)** Relative protein expression levels for MPO, NE, and Cit-H3 normalized to GAPDH, respectively. All three markers were significantly increased in Delta-infected cats. **(E-G)** Quantitative RT-PCR demonstrating mRNA expression levels of MPO, NE, and histone H3 (H3), normalized to GAPDH, respectively. mRNA levels were significantly upregulated in MPO of Delta-infected cats compared to controls and H3 compared to Omicron-infected cats. Statistical interpretations were performed using one-way ANOVA. Data are presented as mean ± SEM; statistical significance is indicated by asterisks: *p < 0.05, **p < 0.01, ****p < 0.0001.

The expression levels of myeloperoxidase (MPO), neutrophil elastase (NE), and citrullinated histone H3 (Cit-H3) were assessed through western blot (**Figure 8A**) and qRT-PCR analyses across three groups: Protein levels of MPO (**Figure 8B**), NE (**Figure 8C**) and citrullinated H3 (**Figure 8D**) were significantly increased in the Delta-infected cats compared to controls (p < 0.01), while the Omicron-infected cats showed markedly lower levels that were not significantly different from controls. mRNA expression of MPO (**Figure 8E**) was significantly elevated in the Delta-infected cats compared to controls (p < 0.05). While the mRNA expression of NE was not significantly higher in the Delta-infected cats (**Figure 8F**), mRNA levels of H3 (**Figure 8G**) were significantly increased in the Delta-infected cats compared to Omicron-infected cats (p < 0.0001).

### 3.7 Elevated MPO-DNA complexes and cell-free DNA are present in Delta variant-infected cats

**Figure 9:**
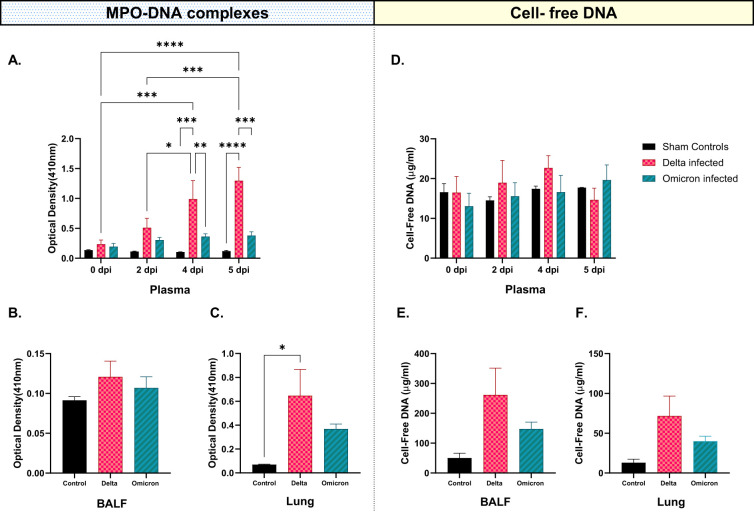
Concentrations of MPO-DNA complexes and cell-free DNA in blood, BALF, and lung tissues obtained from all cats over time. **(A)** MPO-DNA complexes in plasma **(B)** MPO-DNA complexes in BALF and **(C)** MPO-DNA complexes in lung tissue revealed significant alterations in MPO-DNA complexes favoring Delta-infected cats at 2, 4, and 5 dpi, as compared to the controls and Omicron-infected cats. Similarly, **(D)** cell-free DNA in blood, **(E)** cell-free DNA in BALF, and **(F)** cell-free DNA in lungs were altered majorly in Delta-infected cats compared to Omicron-infected cats and sham-inoculated controls. The statistical analyses were performed using two-way and one-way ANOVA. Data are represented as mean ± SEM (*p < 0.05, **p < 0.01, ***p < 0.001, ****p < 0.0001).

In this analysis, MPO-DNA complexes were quantified using MPO-DNA ELISA, and the cell-free DNA was quantified using quantipico dsDNA assay in plasma, BALF, and lungs of all cats. Plasma samples were obtained at 0-,2-,4-, and 5 dpi, while BALF and lung samples were obtained at 5 dpi. MPO-DNA complexes in plasma (**Figure 9A**) were elevated in both variants across all time points, with Delta exhibiting the highest -concentrations at 4 dpi and 5 dpi (p < 0.001, p < 0.0001) compared to controls. At 5 dpi, while Delta remained elevated, Omicron showed a slight decrease, yet higher than controls. Notably, MPO-DNA complexes were significantly increased in Delta-infected cats compared to Omicron-infected cats at 4 dpi and 5 dpi (p < 0.01, p < 0.0001). Within the Delta-infected group, MPO-DNA levels continued to increase with a significant increase at 4 dpi and 5 dpi compared to 0 dpi and 2 dpi. MPO-DNA complexes in BALF (**Figure 9B**) and lung (**Figure 9C**) of both variants were increased compared to controls with a significant increase in Delta-infected lungs compared to controls (p < 0.05) at 5 dpi. Cell-free DNA levels in plasma (**Figure 9D**) exhibited a general increase in both variants with no significant changes at any time point. Similar to plasma, there was a notable increase in cell-free DNA levels in BALF (**Figure 9E**) and lungs (**Figure 9F**) in both Delta and Omicron-infected cats compared to controls at 5 dpi.

### 3.8 Enhanced expression of NETs-related markers in the lung and BALF samples of delta-infected cats

**Figure 10:**
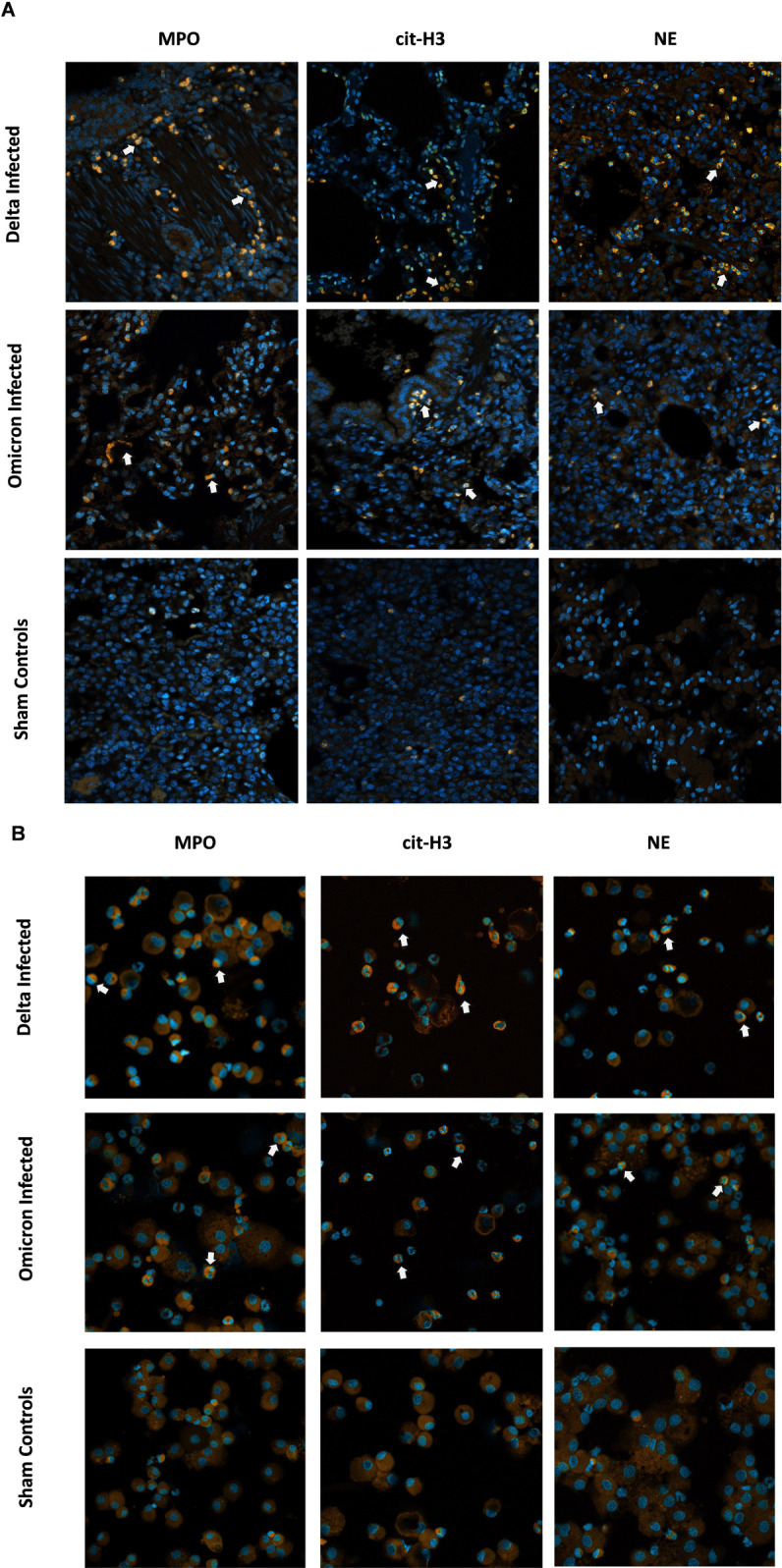
Visual representation of NETs-related markers in lung tissue and BALF in cats following SARS-CoV-2 Infection. Immunofluorescence staining was performed on lung tissues and BAL cells from all cats localizing myeloperoxidase (MPO), citrullinated H3 (cit-H3), and neutrophil elastase (NE). **(A)** Immunofluorescence images from lung tissues of cats infected with SARS-CoV-2 variants of concern (Delta or Omicron) compared to sham-inoculated controls. **(B)** Immunofluorescence images from cytospin slides of BAL from the same groups. DAPI was used as the nuclei counter stain highlighting DNA in blue while MPO/NE/citrullinated H3 was stained in orange. White arrows indicate active neutrophils potentially involved in NETs formation. Magnification **(A, B)**: 20x, scale bar = 50 µm.

Immunofluorescence images of lung tissues (**Figure 10A**) show prominent NET formation (indicated by white arrows) in Delta-infected cats compared to Omicron-infected and control groups. Co-localization of MPO, cit. H3, and NE as indicated by positive staining suggests increased neutrophil activity in the lungs of Delta-infected cats. In contrast, controls and Omicron-infected cats exhibited minimal to moderate fluorescence, indicating either an absence or a significant reduction in neutrophil activation and NET formation. Similarly, immunofluorescent images of BALF samples (**Figure 10B**) from Delta-infected cats demonstrated intensified staining for all three markers: MPO, cit. H3 and NE compared to Omicron-infected cats and controls, emphasizing marked neutrophil activation and potential NETs formation. Omicron-infected cats displayed comparatively lower yet active neutrophil activity in the BALF.

### 3.9 Flowcytometric analysis revealed distinct populations of interest in delta and omicron-infected cats compared to controls

**Figure 11:**
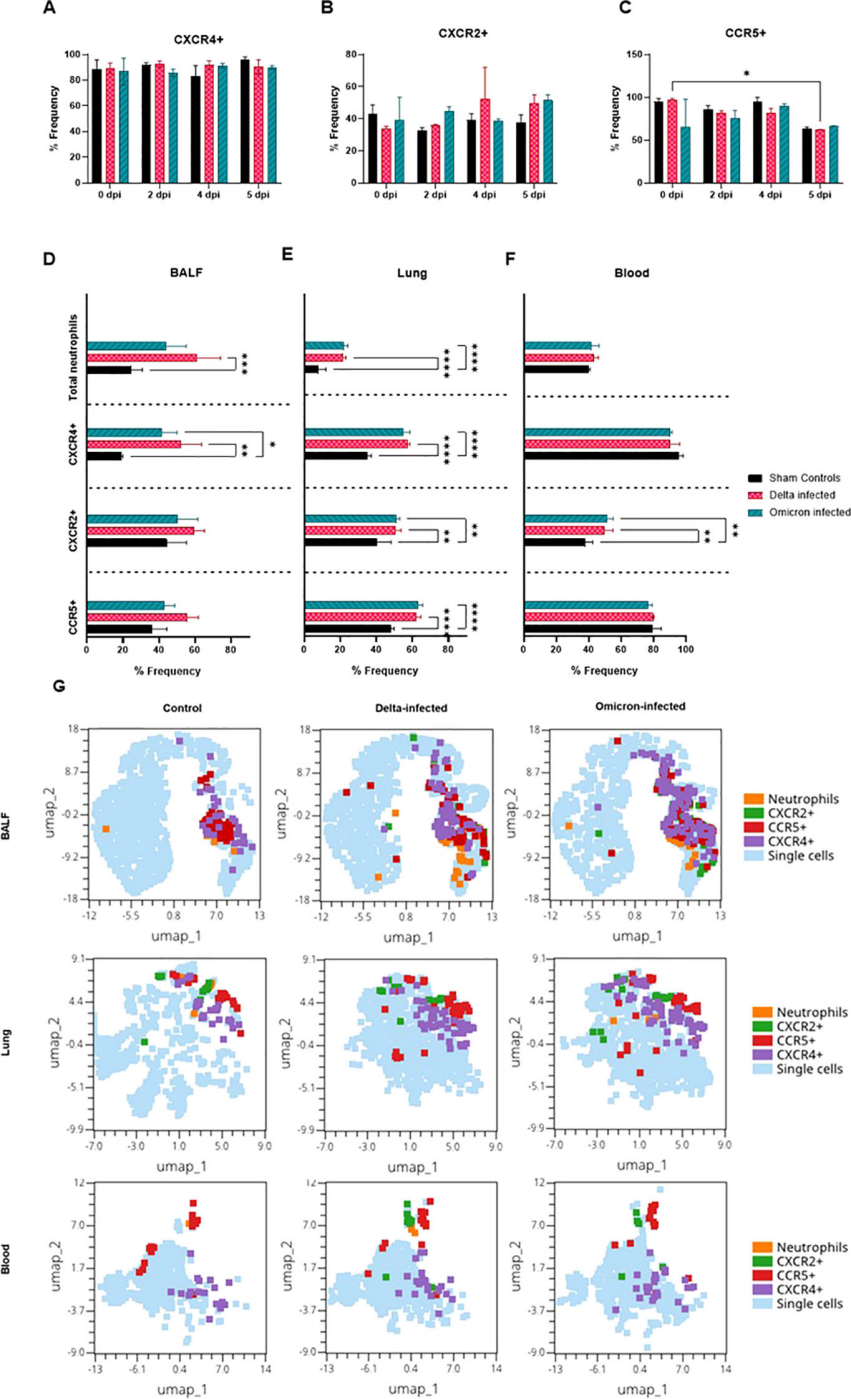
Flow cytometric analysis of neutrophil subsets across blood, BALF, and lung tissues in SARS-CoV-2 infected cats (Delta, Omicron) and controls. **(A-C)** Bar charts represent neutrophil subsets in blood expressing **(A)** CXCR4+, **(B)** CXCR2+, and **(C)** CCR5+ at different time points (0, 2,4, 5 dpi for Delta and Omicron-infected cats compared to controls. **(D-F)** CXCR4+, CXCR2+, CCR5+, and total neutrophils in BALF, lung tissues, and blood at 5 dpi, respectively. **(G)** UMAP plots showing the clustering of neutrophil subsets (CXCR4+, CXCR2+, CCR5+) in BALF, lung, and blood, with different subsets highlighted in respective colors (neutrophils, CXCR2+, CCR5+, CXCR4+). Statistical significance is indicated for key findings using two-way and one-way ANOVA and data are represented as mean ± SE (*p < 0.05, **p < 0.01, ***p < 0.001, ****p < 0.0001).

Flow cytometric analysis revealed notable alterations in neutrophil subsets expressing CXCR4+, CXCR2+, and CCR5+ across blood, BALF, and lung tissues in cats infected with either the Delta or Omicron variants of SARS-CoV-2 compared to sham-inoculated controls. In the blood, while there was no notable change in CXCR4+ subsets (**Figure 11A**), alterations were observed in CXCR2+ subsets (**Figure 11B**), with an increase peaking at 4 dpi in Delta-infected cats. For CCR5+ subsets (**Figure 11C**), a marked decrease was observed in Delta-infected cats at 5 dpi compared to 0 dpi. Flow cytometric evaluation of BALF (**Figure 11D**) revealed significant increase in CXCR4+ subset of Delta-infected cats and Omicron-infected cats compared to controls at 5 dpi, with an increase of total neutrophils in Delta-infected cats compared to controls (p < 0.05, p < 0.01, p < 0.001). Flow cytometric analysis in the lung (**Figure 11E**) demonstrated significant increase in CXCR4+, CXCR2+, and CCR5+ subsets of Delta-infected cats and Omicron-infected cats compared to controls, while total neutrophils were increased in Delta-infected cats and Omicron cats compared to controls at 5 dpi (p < 0.05, p < 0.01, p < 0.001, p < 0.0001). Flow cytometric evaluation of blood (**Figure 11F**) revealed significant increase in CXCR2+ subsets of both Delta and Omicron-infected cats compared to controls (p < 0.01). Notably, the most pronounced increase in neutrophils was seen in Delta-infected cats at 5 dpi. Similarly, UMAP plots of BALF (**Figure 11G**) showed a clear shift in the CXCR4+ CXCR2+, and CCR5+ populations during infection. In Delta-infected cats, CXCR4+ cells formed a dense cluster with expanded distribution compared to controls. Similar trends were observed in the Omicron-infected cats, with a pronounced shift of CXCR4+ neutrophils. UMAP plots of lungs (**Figure 11G**) further emphasized the substantial recruitment and clustering of neutrophil subsets in infected lungs. CXCR4+ and CCR5+ neutrophils showed significant clustering in Delta-infected cats. Moreover, CXCR2+ cells displayed broader spatial distribution in the Delta and Omicron-infected cats compared to controls. UMAP plots of blood (**Figure 11G**) showed varying clustering patterns of CXCR2+, CXCR4+, and CCR5+ subsets. CXCR2+ neutrophils were more widely dispersed in Delta and Omicron-infected cats compared to controls.

## 4 Discussion

The current study provides a comprehensive analysis of the differential clinical, pathological, and immunological responses in cats infected with SARS-CoV-2 Delta and Omicron variants. Insights in the feline model offer additional insights into COVID-19 in people. Clinical assessments reveal more severe and rapid disease progression in Delta-infected cats compared to those infected with the Omicron variant as seen through significantly increased clinical parameters including respiratory effort, wheezing, and activity levels, with peak signs at 5 dpi. The parameters most implicated reflect a greater severity of the lower respiratory tract. These findings are consistent with human studies where Delta variant infection has been associated with more severe lower respiratory symptoms, higher risk of hospitalization, and mortality compared to Omicron infection (7, 43–45). In contrast, the Omicron-infected cats exhibit symptoms most related to upper respiratory tract involvement, such as ocular discharge, suggesting variant-specific manifestations of disease severity. The overall milder clinical signs in Omicron-infected cats may be due to the tropism of the variant for the upper respiratory tract, consistent with other *in vivo* animal and human studies associating Omicron with less severe lower respiratory tract involvement (9, 46–55).

Histopathological evaluations further reinforce clinical findings, highlighting significant variations in tissue damage between the Delta and Omicron variants. While both variants resulted in pathology in the lower respiratory tract and lymphoid tissues, cats infected with Delta showed more severe tissue damage. Cats infected with the Delta variant demonstrated more pronounced lung damage, characterized by thickening of the septa, collapse of alveoli, and substantial inflammatory infiltration, indicating that Delta infection led to significant lung injury and impaired respiratory function. In contrast, Omicron-infected cats demonstrate mild to moderate lung pathology, with mild septal thickening and moderate inflammatory infiltration, indicating a more localized and less severe lung injury. Gross pathology further corroborated these findings. Delta-infected lungs appear severely consolidated with significant congestion, hemorrhage, and necrosis while Omicron-infected lungs show more localized congestion and less extensive damage. Interestingly, both variants cause significant pathology in the upper and middle respiratory tracts, with Omicron infection exhibiting a higher fold change in nasal turbinate and distal tracheal pathology compared to Delta, which is consistent with clinical manifestations of Omicron in other animal models and humans, often characterized by sore throat and nasal congestion (9, 10, 51, 56–59).

The distinct clinical and pathological outcomes observed between the variants may be partly explained by differences in immune responses. Cytokine profiling in both plasma and BALF reveal heightened proinflammatory responses in Delta-infected cats compared to Omicron-infected and control cats. IL-1β was excluded from plasma analysis, and IL-2 was excluded from the BALF analysis as their concentrations were beyond the detectable range. Delta-infected cats exhibited elevated levels of key cytokines including IL-6, IFN-γ, TNF-α, and IL-8, suggesting a more robust inflammatory response. These cytokines are known mediators of acute inflammation and have been associated with severe COVID-19 cases in people (60, 61). Other cytokines, including MCP-1, IL-12, IL-13, SCF, and SDF-1, were also significantly elevated in Delta-infected cats, suggesting a broader activation of immune pathways. Conversely, the cytokine response in Omicron-infected cats was less pronounced, consistent with their milder disease progression. Notably, IL-13, PDGF, and GM-CSF were elevated in Omicron-infected cats, suggesting a skew towards a Th2-type immune response associated with localized inflammation and tissue remodeling (62) rather than the intense systemic inflammation seen with Delta. Within-group analyses of cats infected with Delta indicated a gradual increase in cytokines including IL-18, MCP-1, and IL-6, implying that the immune response in these cats intensifies overtime, which likely plays a role in severe clinical outcomes observed. Furthermore, marked increase of IL-8 and RANTES in Delta-infected cats, with IL-8 serving as a key chemokine for neutrophil recruitment (63) and RANTES playing a pivotal role in platelet activation and thrombosis respectively (64), suggests enhanced neutrophil activity and vascular inflammation likely contributing to the severe pathology observed in Delta-infected cats.

Transcriptomic analyses further highlight the differential molecular responses induced by the two variants. GO and KEGG pathway enrichment analyses identified key pathways involved in neutrophil activation, protein transport, and innate immune responses in Delta-infected cats. Delta infection upregulated genes involved in neutrophil migration, immune response to viral stimuli, and MAPK signaling pathways, indicating excessive cellular stress and inflammatory signaling (65–67). In contrast, Omicron-infected cats show enrichment in processes related to mRNA splicing, response to stress, and activation of innate immune responses. This suggests that while Omicron still triggers an immune response, the cellular mechanisms are more focused on regulating mRNA processing and responding to stress, rather than inducing extensive inflammation (3). GO terms such as protein localization to the endoplasmic reticulum and stress granule assembly suggest that cells in Omicron-infected cats might be engaging in more protective or homeostatic responses to infection. KEGG pathways enriched in Omicron-infected cats including T-cell receptor signaling, PI3K-Akt signaling, mTOR signaling pathways, and activation of PD-L1 suggest an immune-modulating response, which might prevent excessive inflammation, contributing to the reduced severity of lung pathology and clinical symptoms in Omicron-infected cats. Importantly, the comparison between Delta and Omicron-infected cats highlights the key differences in their immune activation profiles. Delta infection led to the enrichment of GO terms related to chronic inflammatory responses, cellular motility, and oxidative stress, which are all critical in the pathogenesis of severe lung injury and sustained inflammation. Moreover, the chronic inflammatory response is particularly relevant, as it emphasizes the ongoing immune activation even after the acute infection, a hallmark of long COVID (68–70). The significant upregulation of genes involved in cell adhesion and immune cell migration in Delta-infected cats further supports the notion of prolonged immune activity, where the continual recruitment of immune cells could contribute to tissue damage and fibrosis seen in long-term COVID cases.

A major finding in this study is the elevated neutrophil activation and formation of NETs in Delta-infected cats compared to Omicron-infected cats. Delta-infected cats show elevated total neutrophil counts, banded neutrophils, and neutrophil-to-lymphocyte ratios (NLR), both in blood and BALF, compared to Omicron-infected cats. This indicates systemic and local recruitment of neutrophils to the site of infection. In plasma, the persistent elevation of neutrophil counts over multiple time points in Delta-infected cats suggests sustained inflammatory responses, often associated with more severe disease progression (14, 16). Moreover, the significant increase of banded neutrophils in Delta-infected cats indicates the release of immature neutrophils from the bone marrow, a hallmark of acute and severe infection which reflects the attempt of the body to rapidly replenish its neutrophil reserves to continue fighting the virus. Moreover, the neutrophil-to-lymphocyte ratio (NLR) is a well-established indicator of systemic inflammation and disease severity in viral infections (71). The elevated NLR observed in Delta-infected cats reflects a predominance of neutrophil-driven inflammation compared to lymphocyte-mediated responses, which may contribute to the significant lung pathology and clinical deterioration seen in these cats. In contrast, the less pronounced increase in NLR in Omicron-infected cats suggests a more balanced immune response, potentially contributing to their milder disease course. This finding is consistent with reports from human studies, where Delta variant-related infections correlate with higher NLRs and more severe disease, while Omicron infections generally result in lower NLRs and less severe outcomes (72–74). Similarly, the increase in total neutrophils, banded neutrophils, and NLR in BALF of Delta-infected cats compared to Omicron-infected cats and controls further supports the notion of excessive neutrophil activation and recruitment to the lungs. This localized increase in neutrophils within the respiratory system highlights the critical role of neutrophils in driving lung inflammation and damage during Delta variant infection. The significant increase in banded neutrophils in BALF suggests ongoing recruitment of immature neutrophils to the site of infection, which can exacerbate lung tissue damage through the release of reactive oxygen species (ROS) and NETs (75, 76).

Significant elevations of MPO, NE, and Cit-H3 in Delta-infected cats, as demonstrated by both western blot and qRT-PCR analyses, underscores the heightened neutrophil activation in response to Delta infection. Upregulation of these markers in both plasma and BALF of Delta-infected cats indicates systemic and localized neutrophil activation, with NET components being released into circulation and actively produced within the lungs (13, 16). Elevated levels of these markers in lung lysates further highlight the intense immune response, where extensive NET formation may contribute to severe tissue destruction (77). In contrast, Omicron-infected cats had much lower expression of these markers in plasma, BALF, and lung tissue, indicating a more contained neutrophil activation and NET formation, resulting in lower lung damage. The presence of Cit-H3 in BALF specifically highlights NET formation in the respiratory tract, which can lead to alveolar damage and impaired gas exchange, contributing to the respiratory symptoms observed in Delta-infected cats (15, 20). RT-PCR further confirmed the increased transcription of NETs-related genes in plasma, BALF, and lung lysates of Delta-infected cats, indicating active neutrophil recruitment and NET formation at both systemic and localized levels. In contrast, the much lower protein and mRNA expression of these markers in Omicron-infected cats reflects a less aggressive neutrophil response, correlating with their milder disease progression. Supporting these findings, immunofluorescence assays revealed similar patterns of elevated NET-related markers in Delta-infected cats. The increased levels of MPO-DNA complexes and cell-free DNA in plasma, BALF, and lung lysates of Delta-infected cats further confirm ongoing NET formation. While NETs are initially protective, their uncontrolled formation can cause tissue damage and exacerbate disease severity. The significant elevation of MPO-DNA complexes in plasma, particularly at 4 and 5 dpi, indicates widespread systemic neutrophil activation. While the elevation in cell-free DNA was less pronounced compared to MPO-DNA complexes, it nonetheless indicates systemic tissue damage. BALF analyses revealed the localized neutrophil activation in the lungs, with Delta infections causing excessive NET formation and lung damage, whereas Omicron infections triggered a milder immune response, correlating with less severe lung pathology. Lung tissues further confirmed the extent of localized tissue damage caused by NETosis in Delta-infected cats, where excessive neutrophil activity contributed to severe lung pathology, compared to the more contained response in Omicron-infected cats.

The flow cytometric analysis of neutrophil subsets in blood, BALF, and lung tissues reveal distinct patterns of neutrophil activation and migration in Delta- and Omicron-infected cats. In the blood, CXCR4+ neutrophils show no significant changes in either Delta- or Omicron-infected cats compared to controls, suggesting that this subset was not actively involved in systemic circulation during the infection. CXCR4 typically retains neutrophils in the bone marrow. Hence, the stable levels in the bloodstream may indicate the receptor involvement to be more localized to tissues, such as the lungs, rather than being a marker of systemic mobilization. In contrast, CXCR2+ neutrophils, a key in promoting migration toward inflammatory sites, were significantly elevated in Delta-infected cats, peaking at 4 dpi. This spike suggests active recruitment of neutrophils to inflamed tissues, such as the lungs, reflecting the aggressive systemic response typical of Delta variant infection. While Omicron-infected cats also show an increase in CXCR2+ neutrophils, it is less pronounced, correlating with their milder inflammatory response. CCR5+ neutrophils, which play a crucial role in leukocyte trafficking, are significantly reduced in the blood of Delta-infected cats by 5 dpi. This reduction indicates the migration of these cells to the lungs or other inflamed tissues. In contrast, the decrease in CCR5+ neutrophils is less pronounced in Omicron-infected cats, suggesting milder inflammatory responses. In BALF, both Delta- and Omicron-infected cats show a significant increase in CXCR4+ neutrophils at 5 dpi, with Delta-infected cats also displaying elevated CXCR2+ and CCR5+ subsets, highlighting robust neutrophil recruitment to the lungs. Lung tissue analysis revealed a marked increase in all three neutrophil subsets in Delta-infected cats, consistent with the intense localized immune response and severe lung pathology. The clustering of neutrophil subsets, illustrated in UMAP plots, highlights the substantial recruitment and activation of neutrophils within the lung tissues of cats infected with Delta, contributing to the intense inflammatory response and tissue damage. In contrast, Omicron-infected cats show a less dramatic increase in these neutrophil subsets in the lungs, corresponding with their comparatively reduced lung pathology and less aggressive immune response. The prevalence of CXCR2+ neutrophils, especially in cats infected with Delta, highlights their role in facilitating neutrophil migration and tissue infiltration, which further exacerbates the significant lung damage observed. Similar patterns have been observed in human studies of COVID-19, where elevated CXCR2+ neutrophils in the blood correlate with increased neutrophil infiltration in the lungs and are associated with worse clinical outcomes (78, 79). The decrease in CCR5+ neutrophils in Delta-infected cats mirrors findings in COVID-19 patients, where impaired leukocyte trafficking has been linked to severe inflammatory responses (80).

As the SARS-CoV-2 virus continues to evolve, variant-specific therapies are increasingly essential in managing COVID-19. Current therapeutic strategies include antiviral medications, monoclonal antibodies, and supportive treatments, which have shown varying degrees of effectiveness depending on the circulating variant (1, 67, 69, 81). For instance, therapies targeting the spike protein may be less effective against variants like Omicron, which harbor mutations that reduce neutralization by some monoclonal antibodies. Furthermore, therapeutic interventions aimed at modulating the immune response, including corticosteroids and various anti-inflammatory medications, could provide therapeutic advantages in addressing the severe inflammation linked to variants like Delta. This research provides valuable information that plays a crucial role in guiding the development of targeted treatment approaches tailored to specific variants of concern. By elucidating the distinct immunological profiles associated with Delta and Omicron infections in feline models, the research highlights the need for tailored interventions that address the unique mechanisms of neutrophil activation and NETosis driven by each variant, including future variants. For instance, therapeutic approaches aimed at reducing excessive activation of neutrophils or the formation of NETs could be particularly beneficial for individuals infected with the Delta variant of SARS-CoV-2, given the significant contribution of these mechanisms in the onset of severe lung injury and systemic inflammation linked to the infection. Conversely, understanding the milder immune response associated with Omicron may lead to more conservative treatment strategies that avoid over-inhibition of the immune system, which could compromise the host immune response against the virus. Moreover, the comparative analysis of neutrophil responses across variants underscores the potential for developing biomarkers to predict disease severity and therapeutic response. Ultimately, this research highlights the crucial need for dynamic and adaptive treatment strategies that consider the constantly evolving landscape of SARS-CoV-2 variants, ensuring effective management of COVID-19 in both human and animal populations.

Importantly, the utilization of feline models in studying SARS-CoV-2 is pivotal for enhancing our understanding of the impact of SARS-CoV-2 virus on both animal and human populations. Cats are an excellent model for exploring viral pathogenesis and immune responses due to their physiological and genetic similarities to humans. This research emphasizes the importance of recognizing variant-specific immune responses in cats, especially in light of their status as companion animals. The relatively mild symptoms associated with the Omicron variant in cats could hinder the detection of infections, emphasizing the necessity for careful monitoring of cats for SARS-CoV-2. Given their potential to act as reservoirs and possible transmitters of the virus, a comprehensive understanding of their immunological responses is crucial for guiding strategies related to public health and infectious disease management. Despite the significant findings of this study, several limitations should be acknowledged. The relatively small sample size limits the generalizability of the results, particularly in the context of natural variations in immune responses among individual cats.To further strengthen and validate these preliminary findings, we emphasize the critical need for subsequent studies involving larger animal cohorts, which would enable more robust statistical analyses, facilitate comprehensive evaluation of biological variability, and improve the translational applicability of the feline model to human disease. Wild-type SARS-CoV-2 is not included in this analysis but would provide a more comprehensive understanding of the immune dynamics and pathogenicity associated with each variant compared to the original strain. Furthermore, the research primarily focused on short-term outcomes, with the long-term effects of infection by either variant remain to be explored. In addition, although using a feline model provides valuable insights, caution is warranted when extrapolating these findings to human infections due to species specific variations in immune responses.

In conclusion, our research underscores the importance of understanding the variant specific immune response and the underlying pathophysiological mechanisms associated with SARS-CoV-2 infection. Importantly, the similarities in immune responses and disease processes observed in cats and humans suggest that felines could serve as an ideal animal model for COVID-19 research, providing valuable insights that can inform human health strategies. Our findings elucidate the variant-specific pathogenesis of SARS-CoV-2, particularly demonstrating that Delta infection leads to more severe clinical and immunological effects compared to those caused by the Omicron variant. This difference carries significant implications for understanding variant-specific disease manifestations and developing tailored therapeutic interventions. Specifically, our study indicates that targeting neutrophil activation and the NETs production may offer a promising therapeutic strategy to mitigate severe lung damage in Delta variant cases. Conversely, the reduced immune activation noted in cats infected with the Omicron variant suggests the requirement of distinct therapeutic modalities in managing Omicron infection, potentially focusing on enhancing viral clearance while minimizing the risk of heightened inflammation.

## Data Availability

The datasets presented in this study can be found in online repositories. The names of the repository/repositories and accession number(s) can be found below: https://www.ncbi.nlm.nih.gov/, PRJNA1186096.
