# Supplementary material for: Comparative analysis of neutrophil dynamics and disease in SARS-CoV-2 Delta and Omicron variants utilizing an in vivo feline model for COVID-19
Source: Front Immunol. 2025 May 22;16:1547918. doi: 10.3389/fimmu.2025.1547918 (PMC12137312; doi:10.3389/fimmu.2025.1547918)
Supplement: Supplementary file 1 [file Table1.docx]

Supplementary Material

1. **Supplemental Table 1(S1) : Clinical Scoring System for Feline Respiratory Disease**
2. **Supplemental Figure 2(S2): Representative plots for gating strategy used in flowcytometric analysis to define neutrophil populations in whole blood, BAL and lung tissues**
3. Supplemental Figure 3(S3) : Comparison of key histopathologic features in the lung tissue of cats infected with SARS-COV-2 Delta and Omicron variant infection

# Supplemental Table 1(S1) : Clinical Scoring System for Feline Respiratory Disease(1–3).

| **Clinical**  **Score**  **Clinical**  **Parameter** | **0**  **Healthy** | **1**  **Mild** | **2**  **Moderate** | **3**  **Marked** |
| --- | --- | --- | --- | --- |
| **Body Weight** | No weight loss | <5% weight loss | 5 to 10% weight loss | >10% weight loss |
| **Temperature** | 37.2 to 39.0°C | 39.1 to 39.4°C | 39.5 to 39.7°C | >39.7°C |
| **Pulse Oximetry** | 98 to 100% | 96 to 97% | 93 to 95% | <93% |
| **Activity** | Normal | Mild reduction when disturbed* (mild lethargy) | Moderate reduction when disturbed* (moderate lethargy) | Little to no activity disturbed* and reduced activity when stimulated** |
| **Behavior** | Normal | Noticeable, but minimal reduction in interest in food and/or attention | Moderate reduction in interest in food and/or attention | Anorexia and/or complete lack of interest |
| **Respiratory Effort** | Normal resting respiratory effort and rate | Mild tachypnea (>35 breaths per minute at rest) with no overt increase in respiratory effort | Moderate tachypnea (>40 breaths per minute at rest) with moderate increase in effort | Marked tachypnea (>45 breaths per minute at rest) with marked dyspnea or effort |
| **Ocular and/or Nasal Discharge** | None | Mild discharge observed from nares or eyes | Moderate discharge observed from either nares or eyes or from both nares and eyes | Marked or purulent discharge noted from nares and/or eyes |
| **Coughing** | None | Occasional, rare cough observed | Intermittent coughing (at least one episode per 30 min) | Marked, persistent coughing (2+ episodes per 30 min) |
| **Wheezing** | None | Occasional, rare wheeze observed | Intermittent wheezing (at least one episode per 30 min) | Marked, persistent wheezing (2+ episodes per 30 min) |

**
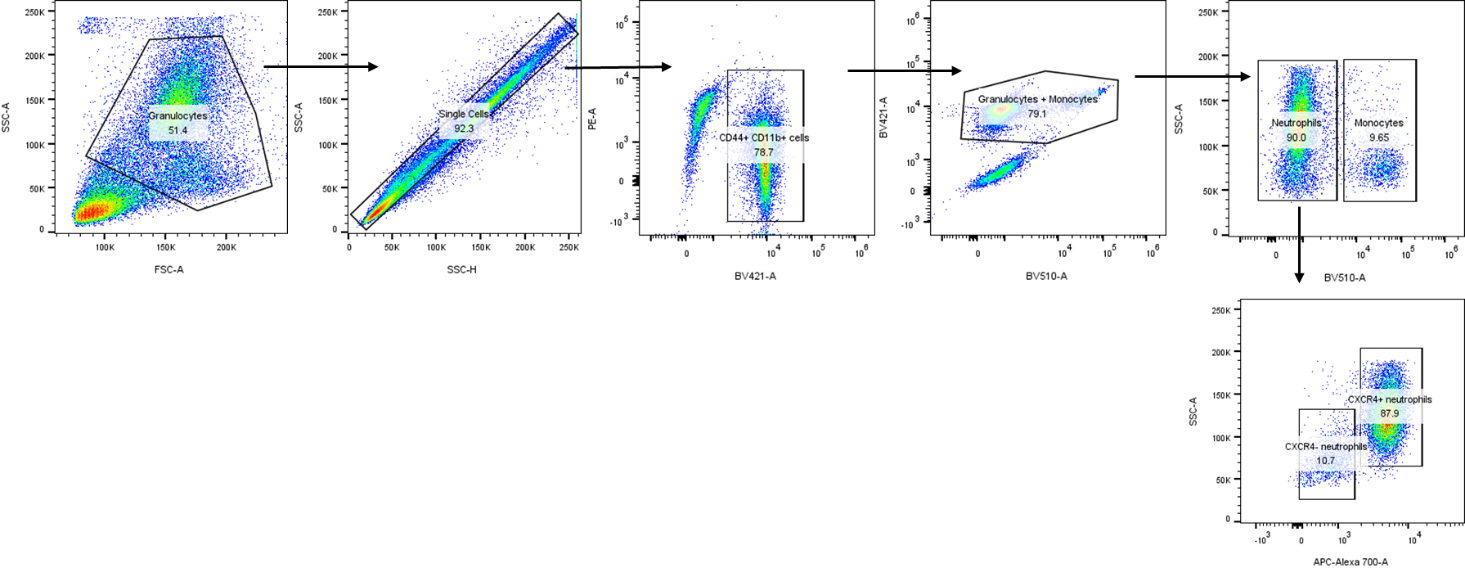
**

**Supplemental Figure 2(S2) :** **Representative plots for gating strategy used in flowcytometric analysis to define neutrophil populations in whole blood, BAL and lung tissues(4).**


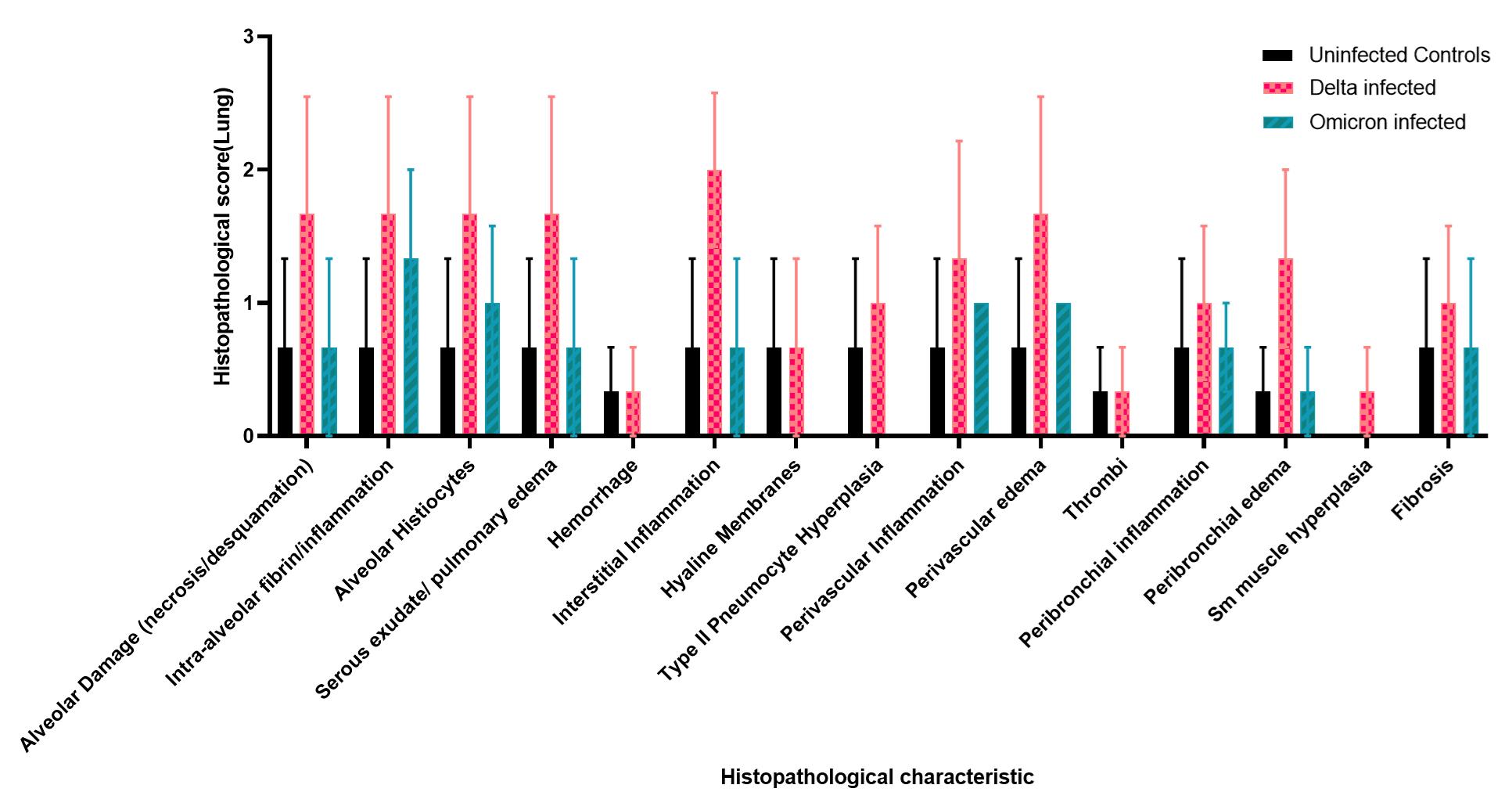


Supplemental Figure 3(S3) : Comparison of key histopathologic features in the lung tissue of cats infected with SARS-COV-2 Delta and Omicron variant infection. The bar graph displays mean scores (± SE) for various histopathologic parameters assessed at multiple time points post-infection including interstitial pneumonia severity, alveolar edema, inflammatory cell infiltration, and bronchiolar epithelial damage. Data indicates notable differences in tissue pathology between the two variants, with Delta generally showing more severe pathology compared to Omicron.
